# Energy cascades in donor-acceptor exciton-polaritons observed by ultrafast two-dimensional white-light spectroscopy

**DOI:** 10.1038/s41467-022-35046-2

**Published:** 2022-11-27

**Authors:** Minjung Son, Zachary T. Armstrong, Ryan T. Allen, Abitha Dhavamani, Michael S. Arnold, Martin T. Zanni

**Affiliations:** 1 Department of Chemistry, University of Wisconsin-Madison, 1101 University Ave, Madison, WI 53706 USA; 2 Department of Materials Science and Engineering, University of Wisconsin-Madison, 1509 University Ave, Madison, WI 53706 USA

**Keywords:** Energy transfer, Excited states, Nanoscale materials

## Abstract

Exciton-polaritons are hybrid states formed when molecular excitons are strongly coupled to photons trapped in an optical cavity. These systems exhibit many interesting, but not fully understood, phenomena. Here, we utilize ultrafast two-dimensional white-light spectroscopy to study donor-acceptor microcavities made from two different layers of semiconducting carbon nanotubes. We observe the delayed growth of a cross peak between the upper- and lower-polariton bands that is oftentimes obscured by Rabi contraction. We simulate the spectra and use Redfield theory to learn that energy cascades down a manifold of new electronic states created by intermolecular coupling and the two distinct bandgaps of the donor and acceptor. Energy most effectively enters the manifold when light-matter coupling is commensurate with the energy distribution of the manifold, contributing to long-range energy transfer. Our results broaden the understanding of energy transfer dynamics in exciton-polariton systems and provide evidence that long-range energy transfer benefits from moderately-coupled cavities.

## Abstract (web summary)

Exciton-polaritons are hybridized light-matter states that exhibit intriguing phenomena that are unobserved in purely excitonic states. Here, the authors elucidate the photophysical mechanism of polariton-assisted long-range energy transfer in carbon nanotubes using two-dimensional white-light spectroscopy and quantum calculations.

## Introduction

Microcavity exciton-polaritons are quasiparticles formed when molecular transitions resonantly exchange energy with light trapped in an optical cavity^1–4^. The marriage of short-lived cavity photons and longer-lived molecular states, two very disparate entities, gives rise to unique properties that are unobserved in purely photonic or purely molecular systems^2,5–7^. Notably, the photon character causes the wavefunction of the system to be spatially delocalized across the cavity, spanning many molecules simultaneously. The delocalized nature of polariton states has been shown to impact a wide range of photophysical and chemical processes^8–18^. One remarkable example is enhanced energy transfer over hundreds of nanometers to microns, as demonstrated in a variety of organic semiconductors and dyes^13,15,19–21^.

Polariton states have wavefunctions that are a linear combination of molecular and photon states. A set of bright eigenstates emerge at energies distinct from those of the molecular states with an energy separation termed the Rabi splitting, whose magnitude reports on the strength of the light-matter coupling. When more than one chromophore is coupled to the cavity photon, a series of dark states also appear in between the energy of the bright polariton states^22^. Characterization of energy flow in polariton cavities will help the understanding of how they impact photophysical and chemical processes, but studying energy flow in strongly coupled polaritonic systems is difficult, because the bright states are short-lived, since they collapse when the light leaves the cavity, and the dark states cannot be directly probed, since they are not optically active^23–27^. Moreover, the electronic transitions of most molecules are broad and ultrafast transient absorption spectra of exciton-polaritons contain contributions from Rabi contractions caused by photoexcitation, making definitive spectral assignments of energy transfer challenging^27–29^.

Exciton-polaritons have mostly been investigated in the limit where the role of the molecular parameters specific to the chromophores is obscured. Because of the broad electronic transitions, very large light-matter couplings are needed to make the bright states energetically well-separated from the molecular states. In contrast, it is well-known from studies on photosynthetic light-harvesting proteins that the energetic disorder of the individual chromophores and the couplings among them are important factors for determining the nature of the bright and dark eigenstates as well as their corresponding dynamics^30–32^. Indeed, the couplings are often comparable to the energetic disorder, creating energetically overlapping eigenstates that are not spectroscopically resolved but have very different wavefunctions. Thus, one might expect energy transfer to depend on both the strength of the light-matter coupling and molecular properties, and the interplay between them.

In this work, we have created a donor-acceptor polariton microcavity suitable for studying the interplay of light-matter coupling and molecular parameters, using thin-film layers of semiconducting single-walled carbon nanotubes (CNTs) with two different optical bandgap energies. Semiconducting CNTs have much narrower linewidths than the electronic transitions of most molecules and their photophysics in films are understood^33,34^, so that new photophysics created by light-matter coupling can be identified. We build microcavities using two CNT layers that are spatially separated by an insulating polymer barrier to limit inter-tube coupling to those of the same bandgap and ensure that energy transfer can only occur due to polaritonic effects^13,15,21^. We utilize two-dimensional white-light (2DWL) spectroscopy so that we can identify energy transfer from cross peaks that lie outside of background signals created by Rabi contraction. We observe a 200-fs growth of a cross peak between the upper- (UP) and lower-polariton (LP) energies. Moreover, the UP and LP relax with distinct lifetimes, which is unobserved in single-bandgap control microcavities. Through fits to the spectra and Redfield theory calculations, we find that the presence of the two distinct bandgaps and intermolecular coupling create a manifold of eigenstates with varying photon character, and hence, lifetimes, unlike in the conventional picture with single bright UP, middle-polariton (MP), and LP states in previously reported donor-acceptor polaritons^13–15^. Energy cascades down this manifold depending on the overlap of the eigenstates, their energy gaps, and the bath frequencies. Our findings shed light on the previously underappreciated role of molecular parameters in governing the photophysics of exciton-polaritons and how energy transfer may benefit from moderately-coupled cavities.

## Results

### 2DWL spectroscopy of CNT microcavities

**Fig. 1:**
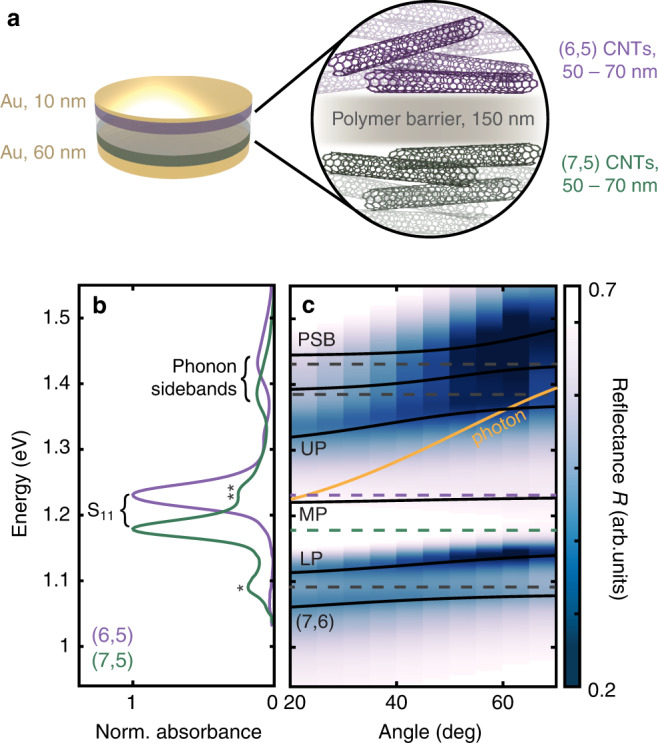
Structure of the microcavity and steady-state spectra. **a** Schematic illustration of the mixed (6,5)/(7,5) carbon nanotube (CNT) microcavity investigated in this work. **b** Linear absorption spectra of the uncoupled CNTs measured in 1,2-dichlorobenzene, each normalized to the S_11_ peak maximum. The two peaks labeled with asterisks denote minor impurities in the (7,5) CNTs from residual (7,6) (*, 1.09 eV) and (6,5) (**, 1.23 eV) chiralities^70^. **c** Angle-dependent reflectance (*R*) spectra of the microcavity shown in **a**. Dashed lines illustrate the uncoupled CNT state energies as shown in **b**, where the energy levels of the two most intense transitions, *i.e*., those that couple most strongly to light, are highlighted in purple ((6,5)) and green ((7,5)). Black solid lines are coupled oscillator model fits to the measured spectra, and the yellow solid line shows the cavity dispersion profile determined from the model (see Supplementary Note 3).

**Fig. 2:**
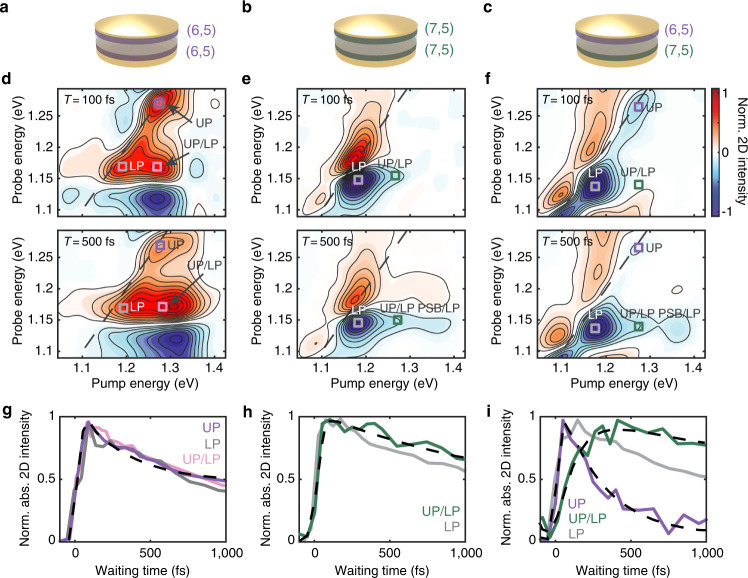
Two-dimensional white-light (2DWL) spectra and energy relaxation dynamics in the microcavities. Cartoon illustrations of the (6,5)- (**a**), (7,5)- (**b**) and mixed (6,5)/(7,5) microcavities (**c**). **d**–**f** 2DWL spectra of the microcavities at waiting times (*T*) of 100 fs (top) and 500 fs (bottom). The spectra are normalized to the maximum magnitude of the 2D signal in the *T* = 100 fs spectrum for each sample. Ground-state bleach/stimulated emission and excited-state absorption are plotted in negative and positive signs, respectively. **g**–**i** Normalized waiting time traces (colored solid lines) generated at the peak positions labeled with open squares in **d**–**f**. Black dashed lines show the fit curves for the upper-polariton (UP) diagonal and UP/lower-polariton (LP) peaks. All traces are plotted in absolute values, i.e., traces for negative peaks are plotted with signs flipped. See also Supplementary Figs. 18–23 for additional data.

Our donor-acceptor microcavity contains semiconducting single-walled CNTs of two different chiralities, known as (6,5) and (7,5), held between a pair of partially reflective gold mirrors (Fig. 1a and Supplementary Figs. 1, 2). The (6,5)- and (7,5) CNTs have different diameters and so exhibit different S_11_ bandgap energies, at 1.23 and 1.18 eV, respectively^35^, thereby serving as the donor and acceptor chromophores, respectively (Fig. 1b). The weaker bands at 1.43 eV and 1.38 eV are phonon sidebands (PSBs) of the (6,5)- and (7,5) CNTs, respectively^36–38^. The overall thickness of our microcavity was designed such that the cavity mode energy falls between the two S_11_ transitions (1.16 − 1.29 eV at normal incidence estimated by a transfer matrix method simulation; see Supplementary Fig. 3 and Methods). We also fabricated single-bandgap control microcavities that are made solely from the (6,5)- or (7,5) CNTs (Fig. 2a, b).

Angle-dependent reflectance spectra were measured to characterize the light-matter coupling (Fig. 1c and Supplementary Fig. 4). Peaks are observed at energies distinct from those of the uncoupled CNT states and shift in position as a function of the incident angle as expected for polaritons. The highest- and lowest-energy eigenstates, labeled PSB and (7,6) in Fig. 1c, respectively, are mostly caused by coupling of the PSB- and (7,6) transitions to the cavity photon. In between, there are three transitions including the two states that are the focus of this manuscript, which we label Upper Polariton (UP) and Lower Polariton (LP). We label the weak transition in the middle the Middle Polariton (MP). The UP and LP result, predominantly, from the coupling of the S_11_ states of the (6,5)- and (7,5) CNTs to the cavity photon that interest us most. A coupled oscillator model was employed to determine the polariton eigenstate energies as well as the cavity dispersion profile, the result of which is shown as black and yellow solid lines in Fig. 1c, Supplementary Figs. 5, 6 and discussed further in Supplementary Note 3.

The excited-state dynamics of the microcavities (Fig. 2a–c) were characterized by 2DWL spectroscopy, a technique that maps out energy transfer through cross peaks with ultrafast time resolution^33^. Fig. 2d, e shows representative 2DWL spectra of the single-bandgap microcavities at *T* = 100 and 500 fs. The spectra exhibit derivative lineshapes with positive and negative features along the probe energy axis. These are characteristic features of Rabi contraction, where the depletion of ground-state population upon photoexcitation by the pump pulse results in a reduction in Rabi splitting, and subsequently a transient shift in peak positions^28,29,39–41^. Thus, the peak energies identified in the 2DWL spectra do not exactly match the energies of the eigenstates identified in the steady-state spectra. The negative and positive features in the 2DWL spectra track the shifts in peak energies due to Rabi contraction, not the peak energies themselves. Based on the peak positions identified in the angle-dependent reflectance spectra and comparing them to the positions of the derivative features in the 2DWL spectra, we assign the diagonal features in each of the spectra to the UP and LP (for the (7,5) microcavity, the UP is pumped but not probed, and so its diagonal peak is not measured, but the UP/LP cross peak is observed).

The samples made solely from single-bandgap CNTs exhibit similar kinetics across the spectral range measured (Fig. 2g, h). All features show a growth within the time resolution of our spectrometer (70 fs) followed by a slow biexponential return to the baseline. To within the error bars, the return to baseline has the same kinetics in both single-bandgap microcavities, with time constants of 1.1 ± 0.2 and 8.5 ± 0.9 ps for the (6,5)- and 0.8 ± 0.2 ps and 7.9 ± 0.8 ps for the (7,5) microcavity, which is consistent with the biexponential kinetics measured in CNT films^42,43^. Thus, to a first approximation, all of the features in the single-bandgap samples have uniform kinetics.

The 2DWL spectra of the mixed (6,5)/(7,5) microcavity (Fig. 2c) are shown in Fig. 2f. In the *T* = 100 fs spectrum, we identify diagonal peaks at energies of 1.27 and 1.14 eV, labeled UP and LP, respectively. The LP diagonal peak appears slightly off the diagonal due to destructive interference with the neighboring positive signal, as commonly occurs in 2D electronic spectroscopy^33^. The peaks are analogous to those in the other two samples; the LP peak resembles that in the (7,5) microcavity, and the UP peak is consistent with that of the (6,5) microcavity. The Rabi splitting between the UP and LP, identified from their peak positions, is 90 meV. While the spectral features are analogous in all three microcavities, the kinetics are distinct between the single-bandgap and mixed microcavities. In the mixed (6,5)/(7,5) sample, the UP and LP diagonal peaks decay on distinctly different timescales. The LP goes back to baseline with time constants of 1.0 ± 0.2 ps and 8.4 ± 0.9 ps (gray trace, Fig. 2i), whereas the UP decays much more rapidly with a time constant of 0.21 ± 0.04 ps, followed by a small-amplitude slow component of 8.2 ± 1.3 ps (purple trace, Fig. 2i). Also different is the appearance of a cross peak with a delayed rise, labeled UP/LP, unlike any other feature in the single-bandgap microcavities. This cross peak grows in on a 200 ± 20 fs timescale (green trace, Fig. 2i). Thus, unlike the microcavities made from single-bandgap CNTs that show uniform dynamics regardless of the feature, the mixed (6,5)/(7,5) microcavity exhibits complex dynamics with different kinetics for nearly every feature.

Given its frequencies and negative sign, we assign the observed cross peak to the stimulated emission of the LP upon photoexcitation into the UP, i.e., the signature for a UP-to-LP energy transfer pathway. We also observe the growth of another cross peak at the pump energy of the PSB and the probe energy of the LP on an identical timescale, indicating that a PSB-to-LP energy transfer step also contributes to the downhill energy relaxation in the microcavity (Supplementary Fig. 23). The cross peak arises from the population rapidly cascading down a manifold of states, populating any one eigenstate for only a few tens of femtoseconds and eventually relaxing into the LP, as will be discussed in detail below. Thus, all downhill transfer pathways from the UP towards the LP contribute to the intensity of the UP/LP cross peak, which is why this particular feature is most sensitive to the energy cascade and little change in lineshape is observed. The observation of energy transfer cross peaks and the extracted sub-picosecond timescale are consistent with recent 2D electronic spectroscopy results on an exciton-polariton microcavity containing TDBC dye aggregates^44^.

Cross peaks typically measure energy transfer^45^. To investigate the origin of the delayed growth of the UP/LP cross peak in the mixed (6,5)/(7,5) microcavity, we performed control experiments in a sample where the cavity mirrors are replaced with plain quartz substrates. No cross peaks are observed, indicating that the cavity mode is required to create this energy transfer pathway (Supplementary Figs. 7, 8). We know that the UP is mostly composed of the (6,5) excitons and the LP mostly of the (7,5) excitons, since the cavity energy sits midway between the bandgaps of the two types of CNTs (Fig. 1c). Thus, we conclude that the cavity enables long-range energy transfer across the insulating barrier as measured by the growth of the UP/LP cross peak. These empirical conclusions are confirmed by classical and quantum mechanical simulations below.

### Modeling of the spectra using the transfer matrix method

**Fig. 3:**
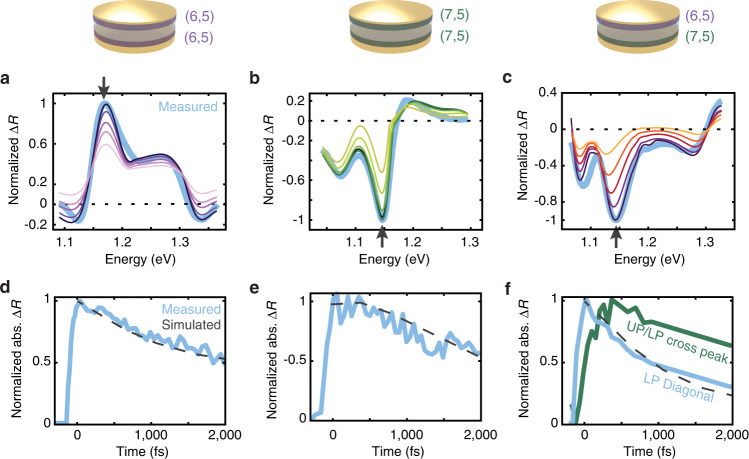
Simulated transient reflection spectra and Rabi contraction dynamics. Transient reflection spectra for the (6,5)- (**a**), (7,5)- (**b**), and mixed (6,5)/(7,5) microcavities (**c**) simulated using the transfer matrix method. Measured transient reflection spectra at *T* = 100 fs are overlaid in light blue for comparison. The dark-to-light color gradient in each panel illustrates the gradual decrease of the bleached population over time (see Supplementary Fig. 15 for the exact values). See Supplementary Note 2.2 and Supplementary Fig. 24 for additional details. **d**–**f** Measured kinetic traces (light blue solid lines) of the lower-polariton (LP) population overlaid with the pseudo-time trace generated from the simulated spectra (black dashed lines). The measured traces are obtained from transient reflection spectra for **d**, **e**, and from two-dimensional white-light (2DWL) spectra for **f** to separately plot the diagonal (light blue) and cross peak (green) contributions. The simulated pseudo-time traces are obtained by taking slices at the wavelengths labeled with black arrows in **a**–**c**. All traces are plotted in absolute values, i.e., traces for negative peaks are plotted with signs flipped.

Absorption/reflection spectra of polaritons are often modeled with classical electrodynamics, using the transfer matrix method^40,46^. We simulated the ground-state (i.e., pump-off) and excited-state (i.e., pump-on) reflection spectra of the microcavities as described in Supplementary Note 2.2. The difference of the ground- and excited-state spectra gives the transient reflection spectra as shown in Fig. 3a–c. These transient spectra are analogous to the 2DWL spectra integrated over all pump energies measured and contain the derivative lineshapes characteristic of Rabi contraction.

As shown in Fig. 3a–c, the model yields exceptionally good agreement with the measured transient reflection spectra, in both the spectral profiles and signs of the signal (see also Supplementary Figs. 18, 19). Because the linewidths are much larger than the frequency shift caused by the Rabi contraction, the percentage of excited population does not appreciably alter the spectral profile. Instead, regardless of how much photoexcitation occurs or how much energy transfers from the UP to the LP, the effect is mostly a change in intensity. Thus, we predict that a Rabi contraction model will display uniform, wavelength-independent kinetics, which is indeed what is observed in the experimental data for the single-bandgap samples (Fig. 3d, e). In contrast, neither the non-monotonic, wavelength-dependent kinetics nor the delayed rise of the UP/LP cross peak observed in the mixed (6,5)/(7,5) sample can be reproduced by this model alone, which only considers energy transfer from the UP to the dark states (Fig. 3f). To explain the kinetics of the mixed (6,5)/(7,5) microcavity, additional states and energy transfer pathways need to be considered. We also note that the cross peak in the mixed cavity does not have derivative lineshapes and so is not spectroscopically consistent with Rabi contraction.

### Modeling of energy transfer using Redfield theory

**Fig. 4:**
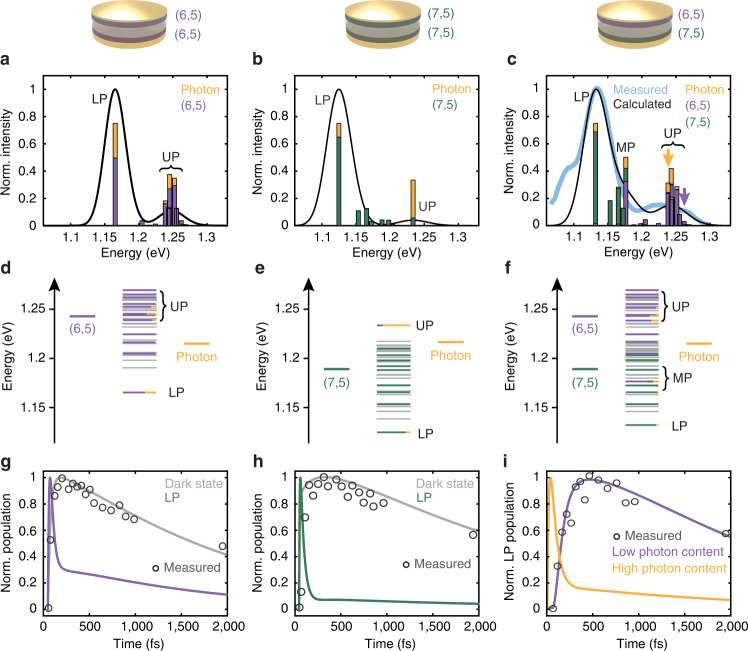
Calculated spectra, energy levels, and dynamics of the microcavities. Calculated linear 1 − *R* spectra of the (6,5)- (**a**), (7,5)- (**b**) and mixed (6,5)/(7,5) (**c**) microcavities. In **c**, the diagonal slice from the measured two-dimensional white-light (2DWL) spectrum in Fig. 2f, top (light blue line) is overlaid with the calculated spectrum (black line) with *J* = − 10 meV and *g* = 42.9 meV, which yielded the best match. These *J* and *g* values were then applied to generate the spectra for the single-bandgap microcavities shown as black lines in **a**, **b**. The vertical bars plot the individual eigenstates with heights proportional to the transition dipole strength, color coded to illustrate the composition of each eigenstate. **d**–**f** Energy level diagrams. Bright states are shown with color codes as in **a**–**c**, and dark states (states with transition dipoles smaller than 0.01% of the maximum dipole strength in each case) are shown in gray. **g**–**i** Calculated population dynamics upon photoexcitation of the upper-polariton (UP) band. In **g**, **h**, the gray and the purple/green traces plot the dark-state and the UP-to-lower-polariton (LP) transfer dynamics, respectively. In **i**, the two traces plot the UP-to-LP transfer dynamics upon pumping states with high- (yellow) and low (purple) photon content, also illustrated as colored arrows in **c**. For comparison, the experimental UP/LP cross peak traces are overlaid as black open circles.

To simulate the spectra and kinetics we have also employed system-bath quantum dynamics calculations using Redfield theory, which considers all possible energy transfer pathways between eigenstates^47–51^. We modeled our system with a Tavis-Cummings Hamiltonian with nearest-neighbor hopping^52–55^:

1 $$\hat{{{{{{{{\mathcal{H}}}}}}}}}=\mathop{\sum}\limits_{i}\hslash {\omega }_{i}\left|i\right\rangle \langle i |+\mathop{\sum}\limits_{i}\mathop{\sum}\limits_{j}J|i\rangle \left\langle j\right |+\hslash \omega _{{{{{{{\rm{c}}}}}}}}{a}^{{{{\dagger}}} }a+\mathop{\sum}\limits_{i}{g}_{i}(\left|G\right\rangle \langle i|{a}^{{{{\dagger}}} }+|i\rangle \left\langle G\right|a)$$

where *ω*_*i*_ is the transition frequency of the *i*th chromophore, *J* is the nearest-neighbor inter-tube coupling, *ω*_*c*_ is the cavity mode energy, *a*^†^ and *a* are photon creation and annihilation operators, and *g*_*i*_ is the light-matter coupling to each CNT. The collective coupling strength *g*_tot_ is given by:

2 $${g}_{{{{{{{{\rm{tot}}}}}}}}}=\mathop{\sum}\limits_{k}{\mu }_{k}\sqrt{\frac{{N}_{k}\hslash {\omega }_{{{{{{{{\rm{c}}}}}}}}}}{2V{\epsilon }_{o}}}$$

where *μ*_*k*_ and *N*_*k*_ are the transition dipole and number of the chromophores of that type (*k* = (6,5) or (7,5)), respectively, and *V* is the mode volume. In what follows, we label *g*_tot_ as *g* for simplicity throughout the text and figures. When cast, the CNTs aggregate into bundles that consist of tens of nanotubes with little-to-no coupling between bundles^34,56^. Thus, the CNTs are modeled as hexagonally packed bundles of 36 nanotubes. Increasing the size or number of the bundles did not impact the conclusions reported below, as shown in Supplementary Fig. 13 and discussed in Supplementary Note 5.6. The nearest-neighbor electronic coupling is introduced only among CNTs of the same bandgaps (*J* = −10 meV)^57–59^, but not between the (6,5)- and (7,5) CNTs due to the presence of the insulating polymer barrier (see Supplementary Notes 5.1, 5.2 and Supplementary Fig. 9 for details of the procedure and associated data). By fitting the calculated linear spectrum to a diagonal slice through the experimental 2DWL spectrum, we determine that the best match occurs when *ω*_*c*_ = 1.217 eV and *g* = 42.9 meV (Fig. 4c). The Rabi splitting estimated from the UP-LP energy gap in the experimental spectrum is 90 meV, which is approximately twice that of the *g* value from the fit.

**Fig. 5:**
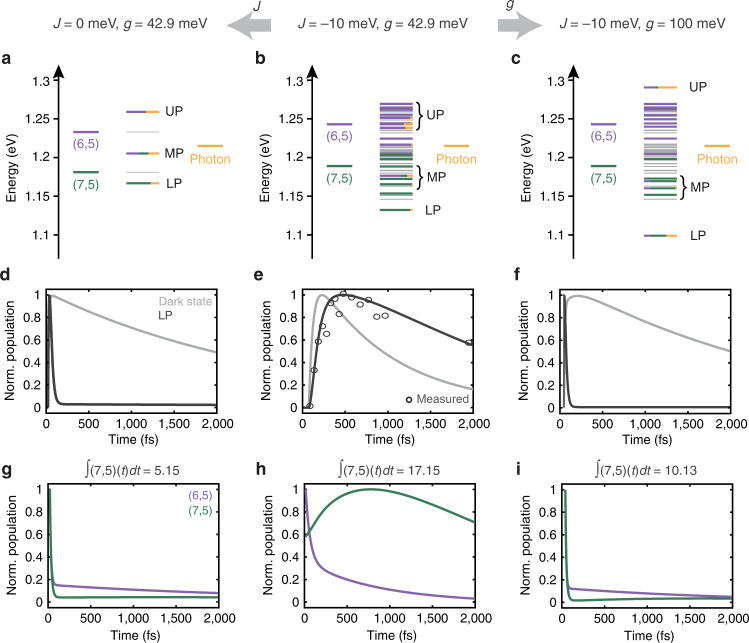
System parameters affecting the energy landscape and population dynamics. Energy level diagrams of the mixed (6,5)/(7,5) microcavity in the absence of inter-tube coupling (*J*) (**a**), when both *J* and light-matter coupling (*g*) are present with comparable amounts (**b**), and when *g* is significantly larger than *J* (**c**). Bright states are color-coded to illustrate the contribution from the cavity photon and the carbon nanotubes (CNTs), and dark states (states with transition dipoles smaller than 0.01% of the maximum dipole strength in each case) are shown in gray. The specific *J* and *g* values used for each case are displayed on top. See also Supplementary Fig. 16 for the simulated spectra. **b** is a replicate of the energy level diagram in Fig. 4c plotted on a different energy axis. **d**–**f** show the corresponding relaxation dynamics of the population upon photoexcitation into the upper-polariton (UP) band. The black and purple traces plot the dynamics of the dark states and the UP-to-lower-polariton (LP) transfer, respectively. The black traces include all transfer pathways that start in the UP band and end in the LP band. The black trace in **e** is a replicate of the purple trace shown in Fig. 4i, and is overlaid with the experimental UP/LP cross peak trace (open circles). **g**–**i** Calculated populations of excited (6,5)- or (7,5) CNTs upon excitation of the UP band. On top of each panel, we show the integrated (7,5) population that results from energy transfer from the (6,5) CNTs.

The simulation of the mixed (6,5)/(7,5) system predicts a myriad of eigenstates with various oscillator strengths (Fig. 4c, f). The eigenstates span about the same energy range as in films of CNT bundles, but have different coefficients. It is the different coefficients of the eigenstates that create the difference in the energy transfer dynamics between films and microcavities. The spectrum can be divided into eigenstates that form the UP, MP, LP, and those that fall in between. The labels are based on the approximate frequencies where one would find UP, LP and MP states in a conventional polariton model (see Fig. 5a below), but we emphasize that they are bands of states that consist of eigenstates that are closely spaced in energy. The eigenstates in both the UP and LP bands have character of both the (6,5)- and (7,5) CNTs, with more (6,5) in the UP and more (7,5) in the LP, as expected from the cavity dispersion profile shown above (Fig. 1c). Many states are dark, with no photon character, while others have small amount of photon character with the maximum photon character in a single eigenstate of 26%. Many of the bright middle states between the UP and LP are predominantly molecular in character with near-zero photon content. The eigenstates are so closely spaced that many cannot be resolved spectroscopically. Thus, pumping the UP band will result in simultaneous excitation of several eigenstates within this band with varying CNT- and photon character. The eigenstates of the single-bandgap samples can similarly be grouped into UP, LP and bright middle states (there is no MP for these systems because there is only one CNT bandgap), but the middle states have far weaker transition dipoles than the bright molecular states in the mixed (6,5)/(7,5) cavity (Fig. 4a, b, d, e).

We simulated the dynamics by evolving the reduced density matrix in time using Redfield theory^49–51^ (see Supplementary Notes 5.1, 5.3, 5.4 and Supplementary Figs. 11, 12 for additional details and data). To mimic the experimental conditions, the dynamics are initiated with all the population residing in the UP band. Fig. 4g-i plots the simulated kinetics of energy flow from the UP to the LP, including all possible transfer pathways that start in the UP band and end in the LP band. We plot the kinetics of the lowest-energy eigenstate in the LP band, because it stands alone and matches the experimental frequency. For the single-bandgap systems, the simulations predict rapid relaxation of the UP to lower-lying states (Supplementary Fig. 14). The LP state relaxes within tens of femtoseconds because it has 34% (6,5) and 14% (7,5) photon character (Fig. 4g, h, purple/green traces). Also plotted are the simulated dark-state dynamics (Fig. 4g, h, gray traces), which match the picosecond dynamics observed in the experiment (Fig. 2g, h). Thus, the fast and slow components of the biexponential decay observed in our 2DWL spectra (Fig. 2g–i) likely correspond to energy cascading down the polariton eigenstate manifold into the LP band and dark-state dynamics, respectively, the latter of which tracks the S_11_ exciton lifetime of the CNTs.

Simulated dynamics of the mixed (6,5)/(7,5) microcavity, in contrast, recover the initial growth of the LP population observed in the experimental cross peak under certain conditions. When an eigenstate in the UP band with high photon content is initially pumped, the dynamics are similar to those for the single-bandgap microcavities. The LP instantaneously grows in following rapid relaxation from the UP and mostly decays within the first 500 fs due to its non-negligible (8%) photon character (Fig. 4i, yellow trace). On the other hand, initial pumping of an eigenstate with low photon content results in strikingly different dynamics (Fig. 4i, purple trace). There is a slow buildup in the LP population over the first 500 fs, followed by a several picosecond decay to the ground state. Kinetics associated with low photon content shows excellent agreement with the measured UP/LP cross peak dynamics (Fig. 4i, circles). There are many other pathways by which energy can relax through this dense manifold in addition to what is shown in Fig. 4i. Supplementary Fig. 10 plots the kinetics of the LP band after excitation of various other initial eigenstates. We also note that inclusion of different **k**-vectors and higher cavity modes will result in a different manifold of eigenstates and correspondingly different relaxation pathways^60^.

Using the kinetics from Redfield theory, we return to the classical Rabi contraction model discussed earlier. If the dark-state populations obtained from Redfield theory (Supplementary Fig. 15) are used to scale the bleach terms in the transfer matrix method simulations, then the measured LP dynamics of the two single-bandgap microcavities are recovered nearly perfectly (Fig. 3d, e) as are the LP diagonal dynamics of the mixed (6,5)/(7,5) cavity (Fig. 3f, light blue trace). However, no features from this classical model mimic the 200-fs growth of the UP/LP cross peak (Fig. 3f, green trace). Thus, we conclude that transient reflection spectra and the diagonal features in the 2DWL spectra mostly reflect Rabi contractions and, hence, exhibit dynamics that mirror the dark-state populations, whereas the cross peak in the 2DWL spectra directly measures energy transfer between states.

## Discussion

Redfield theory allows us to understand how the presence of the two distinct bandgaps as well as inter-tube coupling (*J*) creates a manifold of optically bright states through which energy cascades. We find that cascading occurs when these parameters are present in magnitudes that are comparable to the light-matter coupling (*g*). To illustrate this point, we adjust the best-fit parameters of the Hamiltonian in Equation (1) to explore two different limits, one in which we decrease the inter-tube coupling (Fig. 5a) and the other in which we increase the light-matter coupling (Fig. 5c). In the black traces of Fig. 5d–f and Fig. 5g–i, we plot the simulated population dynamics of the LP and the (6,5)/(7,5) CNTs, respectively. We also show the integrated population of the (7,5) CNTs in each case, which reports on the efficiency of energy transfer across the barrier. 

Figure 5a illustrates the case where no inter-tube coupling is present (*J* = 0 meV). The energy landscape converges into only three well-separated bright states (UP, MP, LP) and degenerate dark states, which is the conventional simple picture used to describe the energetics of exciton-polaritons. Photoexcitation of the UP results in rapid energy transfer to all lower-lying states, i.e., the dark states, MP, LP, and the ground state, due to its sizeable (41%) photon character. Energy that does make it into the dark states within the UP lifetime creates a Rabi contraction (Fig. 5d, gray trace), but in this limit the populations that create cross peaks are extremely short-lived (Fig. 5d, black trace). Fig. 5c illustrates the case where the light-matter coupling is much larger (*g* = 100 meV) than both the inter-tube coupling and difference in the bandgap energies. In this limit, the UP and LP bands appear with sizeable photon character (>31%), in between which a manifold of states with little to no photon character appears. The UP and LP bands are energetically well-separated from the manifold, similarly to the limit shown in Fig. 5a. Once again, the UP decays rapidly, which limits the amount of time for energy to flow into the manifold of molecular states (Fig. 5f, black trace).

On the contrary, when the inter-tube coupling is of comparable magnitude with the light-matter coupling (Fig. 5b), the UP and LP bands are no longer well-separated from the manifold, and the photon content is redistributed over many states within this manifold. As discussed earlier, the LP has only 8% photon character, and so its properties partly resemble those of weakly coupled excitonic states^61,62^. The small energy gap increases the time allotted for energy to enter and cascade down the manifold when a UP state with low photon content is initially excited, creating the delayed rise of the cross peak in the experiment, corresponding to long-range energy transfer from the (6,5)- to the (7,5) layer as illustrated in the growth in (7,5) population (Fig. 5h). The integrated population of the (7,5) CNTs is also the highest, indicating that the energy transfer is enhanced in the moderate coupling case (Fig. 5g–i). Within the linewidth of the UP band, one cannot resolve eigenstates with large versus small photon character, and so the experimental dynamics reflect a convolution of contributions from states with varying photon character.

In summary, we have studied the role that molecular parameters play in the formation and dynamics of exciton-polaritons. Intermolecular couplings or differences in bandgaps create manifolds of closely-spaced, but non-degenerate eigenstates, many of which are bright states with varying photon character. Energy can cascade through these states from the UP to the LP, which are themselves bands of states, creating a cross peak in the 2DWL spectra that exhibits a delayed rise with a ~200 fs time constant. The transient reflection spectra and the diagonal peaks in the 2DWL spectra are dominated by Rabi contractions, but the cross peak in the 2DWL spectra helps resolve specific transfer pathways. We note that the kinetics of the mixed (6,5)/(7,5) microcavity reported here is accompanied by energy transfer across the 150-nm insulating barrier, which is significantly longer than the typical exciton diffusion length of ~10 nm in most organic semiconductors^63^. Our simulations reproduce all of our experimental data and indicate that long-range energy transfer is enhanced by a manifold of eigenstates created by a balance between intermolecular couplings, differences in bandgap, and light-matter coupling, unlike in the conventional picture describing the energy landscape of donor-acceptor polaritons with single discrete UP, MP, and LP states. If the light-matter coupling is much smaller or larger than the energy distribution of the manifold, then long-range energy transfer is less efficient, because the states collapse more quickly than the energy can enter the manifold. In biology, energetic disorder plays a central role in long-range energy transfer. Thus, it may be advantageous to engineer a manifold of states into exciton-polaritons and thereby manipulate both their dynamics and spatial energy flow as often occurs in biological systems. In the case of CNTs, changes in the film thickness or alignment^64^ would provide additional means to control the interplay between intermolecular coupling and light-matter coupling in the system, thereby contributing additional insights into the photophysics and, in turn, design parameters for optimal CNT polaritons.

## Methods

### Purification of semiconducting single-walled CNTs

Semiconducting singled-walled CNTs enriched in (6,5)- and (7,5) chiralities were isolated from as-produced heterogeneous single-walled CNTs (CoMoCAT SG65i, Sigma Aldrich) following previously reported protocols with minor modifications^65,66^. Briefly, polyfluorene derivatives with different functional groups were employed as wrapping polymers to selectively wrap and isolate the (6,5)- and (7,5) CNTs from the heterogeneous mixture (see Supplementary Note 1 for details of the purification procedure). The concentration of the final (6,5)- and (7,5) CNT suspensions (in 1,2-dichlorobenzene) used for fabrication of all microcavity samples in this work was 50 μg mL^−1^.

### Fabrication of the microcavities

The 10-nm and 60-nm-thick gold mirrors were prepared by thermal evaporation of Au pellets (Kurt J. Lesker) at 0.02 nm s^−1^ on quartz substrates (Electron Microscopy Sciences; 15 mm diameter, 0.15 mm thickness). Prior to evaporation, the glass substrates were cleaned by successive sonication in acetone for 1 h and soaking in 2-propanol at 80 °C for 40 min. For each sample, a pair of half-cavities was fabricated by drop-casting 60 μL of either (6,5)- or (7,5) CNT suspension on the 10-nm/60-nm gold-deposited substrates. The solvent was evaporated by drying the half-cavities overnight. Aggregates of the wrapping polymer and CNT bundles were removed by successive soaking in tetrahydrofuran and then 2-propanol (80 °C, 2 h each). Poly(vinyl acetate) (PVA) polymer beads (Sigma Aldrich) were added to chlorobenzene to a concentration of 40 mg/mL and dissolved by stirring overnight at 60 °C. Immediately before lamination of each microcavity sample, the PVA suspension was spin-cast (2000 rpm, 2 min) on one of the two half-cavities, on top of the drop-cast and washed CNT layer. The two half-cavities were assembled in a home-built compressor tool and laminated in an oven at 110 °C for 3 h^38^. The cavity-less control sample (Supplementary Fig. 7a) was fabricated following the same cleaning, drop-casting, and lamination procedures but with a pair of plain glass substrates. Surface roughness and thickness of each component layer were characterized on an atomic force microscope (Bruker, Icon; see Supplementary Figs. 1, 2). The thickness of the CNT layers and the PVA layer was characterized to be 50–70 nm (CNT) and 150 nm (PVA). The 20-nm variation in the thickness of the CNT layers (50–70 nm) originates from the drop-casting method employed in fabrication, which creates a radial gradient of layer thickness, as reported and discussed in our previous work^38^. The microcavity design was identical in all three microcavities with CNT (50–70 nm)-PVA (150 nm)-CNT (50–70 nm) layers, where for the (6,5)- and (7,5) microcavities both CNT layers were of the same chirality. The thickness of each layer, and thus the total cavity thickness, was maintained across the three systems so that the cavity mode dispersion profile was nearly identical for all three microcavities (Supplementary Fig. 4).

### Angle-dependent reflectance spectroscopy

Steady-state angle-dependent reflectance spectra were measured on a V-VASE variable-angle ellipsometer (J. A. Woollam). White light with transverse electric (TE) polarization was focused into a 0.2-mm spot on the sample position, and the light reflected from the 10-nm gold mirror was collected. The angle of incidence was varied from 20° to 70° in 5° steps with a motorized goniometer.

### 2DWL spectroscopy

Detailed description of the 2DWL apparatus and data processing can be found elsewhere^33^. 2DWL was implemented in a pump-probe geometry in an all-parallel polarization scheme. Briefly, the pump and probe white light was generated by splitting the output of a Ti:Sapphire regenerative amplifier (Spectra Physics, Spitfire; 800 nm center wavelength, 1 kHz, 150 fs pulse duration) into pump and probe arms and then focusing each onto a 4-mm thick yttrium aluminum garnet (YAG) crystal (NewLight Photonics). Following collimation, the pump white light was sent into a prism compressor for removal of unwanted wavelengths including the residual 800 nm fundamental as well as compression of the pulse. The temporal resolution of our 2DWL measurements was ~ 70 fs as characterized by fitting the rise time of a non-resonant transient signal. Two pairs of birefringent wedges mounted on a motorized translational stage (Newport) generated the pump pulse pair and controlled the time delay between the pump pulses, the coherence time^67^. The coherence time was sampled in 0.4-fs steps in the range of 0 − 200 fs. The waiting time (*T*), the time delay between the pump and the probe pulses, was controlled by delaying the probe beam with a retroreflector mounted on a motorized translational stage (Thorlabs). *T* was incremented in steps of 50 fs for *T* = −50 to 500 fs, 100 fs for *T* = 500 to 1000 fs, 1000 fs for *T* = 1000 to 2000 fs, and 2000 fs for *T* = 2000 to 10,000 fs. The pump and the probe beams were focused onto the sample with a 90° off-axis parabolic mirror, and were temporally and spatially overlapped. The angle of incidence on the sample plane was ~5° for the pump and ~30° for the probe. The probe beam reflected off the sample was picked off, collimated, and directed into a spectrograph (Princeton Instruments, SpectraPro 2150i). We note that the pulse polarization is invariant upon reflection, because both the incident and reflected laser beams lie in a plane perpendicular to the sample plane. The pump-on and pump-off probe spectra were recorded shot-to-shot at 1 kHz by chopping the pump beam at 0.5 kHz. The 2DWL spectrum at each *T* was obtained by Fourier transforming the raw data, which were spectral interferograms collected in the probe frequency domain and coherence time domain, along the coherence time axis. Transient reflection spectra were also collected by setting the coherence time to zero. A pump fluence of 2.90 × 10^13^ photons cm^−2^ pulse^−1^ was employed for all 2DWL measurements, which was previously reported to be in the single exciton regime^68,69^ and yielded transient reflection signal intensity linearly dependent on the pump fluence (Supplementary Fig. 19b). Given the 70 fs time resolution mentioned above, we only analyze spectra measured at *T* ≥ 100 fs in this work.

## Supplementary information

Supplementary Information

## Data Availability

The data presented in this work are available from the corresponding author upon request.

## Code availability

The codes used to process and analyze the data presented in this work are available from the corresponding author upon request.
