# Supplementary material for: Energy cascades in donor-acceptor exciton-polaritons observed by ultrafast two-dimensional white-light spectroscopy
Source: Nat Commun. 2022 Nov 27;13:7305. doi: 10.1038/s41467-022-35046-2 (PMC9701200; doi:10.1038/s41467-022-35046-2)
Supplement: Supplementary file 1 — Supplementary Information [file 41467_2022_35046_MOESM1_ESM.pdf]

Supplementary Information

Energy cascades in donor-acceptor exciton-polaritons observed by ultrafast two-dimensional white-light spectroscopy

Minjung Son[1], Zachary T. Armstrong[1], Ryan T. Allen[1], Abitha Dhavamani[2], Michael S. Arnold[2], and Martin T. Zanni[1*]

[1]*Department of Chemistry, University of Wisconsin−Madison, 1101 University Ave, Madison, WI 53706, USA*

[2]*Department of Materials Science and Engineering, University of Wisconsin−Madison, 1509 University Ave, Madison, WI 53706, USA*

[*] zanni@chem.wisc.edu

Supplementary Note 1: Detailed description of the purification procedure of (6,5)- and (7,5)-enriched semiconducting single-walled CNTs

For (6,5)-enriched semiconducting single-walled CNTs, poly[(9,9-dioctylfluorenyl-2,7-diyl)-*alt*-co-(6,6'-2,2'-bipyridine)] (PFO-BPy; American Dye Source, ADS153UV) was used as the wrapping polymer. PFO-BPy powder was dissolved in dry toluene at a concentration of 1 mg/mL by magnetic stirring and heating at 80°C. CoMoCAT SG65i (MilliporeSigma) was then added to the PFO-BPy/toluene solution at a 0.5 mg/mL concentration. The mixture was horn-tip ultrasonicated (Fisher, Model 500, 400W) at 40% amplitude for 15 minutes. The resulting slurries were centrifuged at 300,000 g for 10 minutes for removal of soot and large bundles of CNTs. The top 90% supernatant was collected and filtered through a 5 μm PTFE membrane filter, which removed residual impurities. The pellet was mixed with fresh PFO-BPy/toluene solution and reused for two additional rounds of ultrasonication/centrifugation. The supernatant from all three rounds of centrifugation was combined. The solvent was evaporated in a rotary evaporator. The resultant CNT solids were redispersed in hot tetrahydrofuran (THF, 100°C) and repeatedly centrifuged at 50,000 g to separate the desired polymer-wrapped CNTs, which precipitate as pellets, from free excess PFO-BPy, which remains in the supernatant. After each round of centrifugation, the linear absorption spectra of the supernatant as well as the redispersed pellet in THF were measured to quantify the mass of PFO-BPy and polymer-wrapped CNTs. The centrifugation was repeated until the mass ratio between PFO-BPy and CNTs was close to 1:1.

For (7,5)-enriched CNTs, poly[9,9-dioctylfluorenyl-2,7-diyl] (PFO; American Dye Source, ADS329BE) was used as the wrapping polymer. PFO powder was dissolved in dry toluene at a concentration of 1.5 mg/mL by magnetic stirring and heating at 80°C, and 0.5 mg/mL of CoMoCAT SG65i (MilliporeSigma) was added to the PFO/toluene solution similarly to the (6,5) case. The rest of the sorting/purification steps were identical to the procedure for (6,5)-enriched CNTs.

For both chiralities, the final CNT pellets were dispersed in 1,2-dichlorobenzene to a 200 μg/mL concentration and stored until further use. The suspensions were diluted with 1,2-dichlorobenzene to a final concentration of 50 μg/mL immediately before sample fabrication.

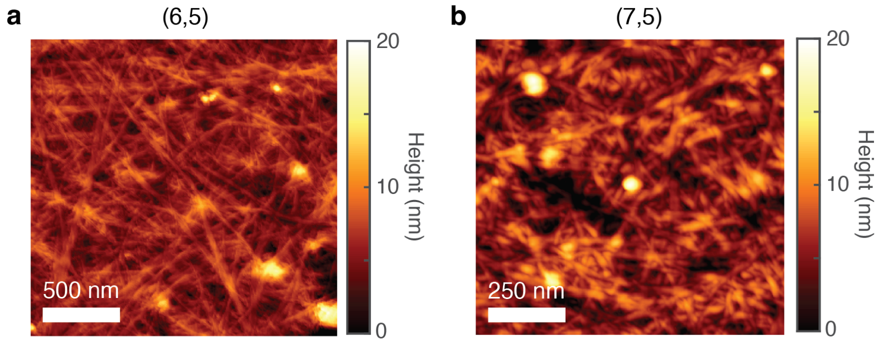

Supplementary Figure 1: AFM images of the CNT layers. Representative AFM images of thin films of (6,5) (**a**) and (7,5) CNTs (**b**) prepared by drop-casting 60 μL of each suspension (50 μg/mL) on quartz cover slips. Scale bars are shown and labeled in each panel.

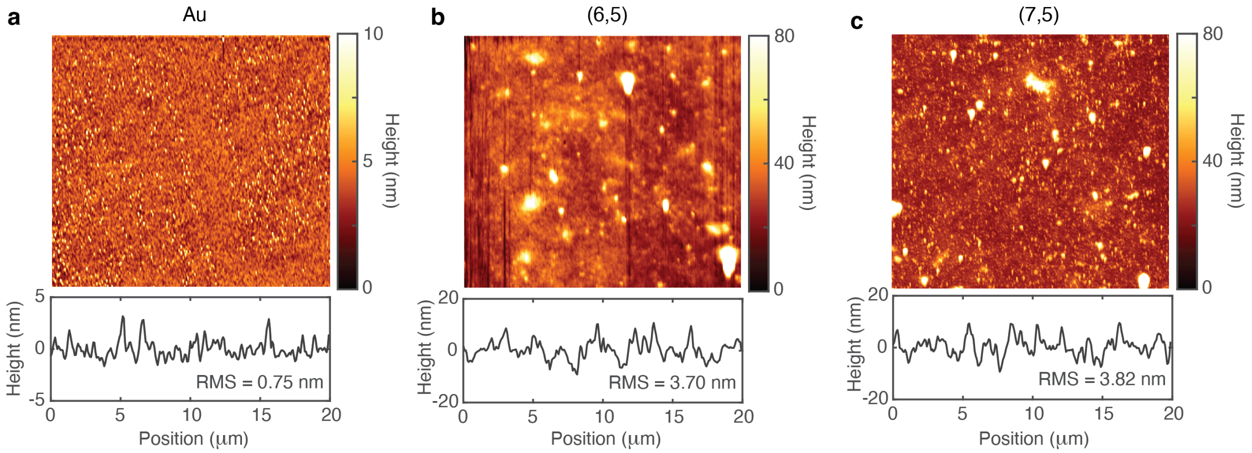

Supplementary Figure 2: AFM characterization of the surface roughness. Representative AFM images of a gold layer (**a**, 60 nm on quartz cover slip), and thin films of (6,5) (**b**) and (7,5) CNTs (**c**) prepared by drop-casting 60 μL of each suspension (50 μg/mL) on cover slips. All three images are 20μm $\times$ 20μm in size. The bottom traces show a representative horizontal slice of the surface profile from each image, with the root mean square (RMS) error indicated.

Supplementary Note 2: Transfer matrix method simulations of the microcavity reflectance spectra

2.1. Simulation of linear reflectance spectra

The reflectance spectra of the microcavities were modeled with the transfer matrix method at each angle of incidence, including at the Brewster angle, at $0-70^\circ$ [1,2]. The optical constants of the CNT layers were obtained from ellipsometry measurements (J. A. Woollam, RC2) of CNT films on quartz substrates prepared in an identical way to how the half-cavities were prepared during microcavity fabrication. The variable-angle reflection and transmission data were fit in the CompleteEASE software using a uniaxial anisotropic model, where the in-plane index was modeled with Cody-Lorentzian/Lorentzian oscillators and the out-of-plane index was modeled with a Cauchy dispersion. In all of our microcavities, transmittance (T) is negligible ($< 1\%$), and so we only consider the reflectance (R) and absorbance ($A = 1 - T - R \approx 1 - R$) spectra throughout this manuscript.

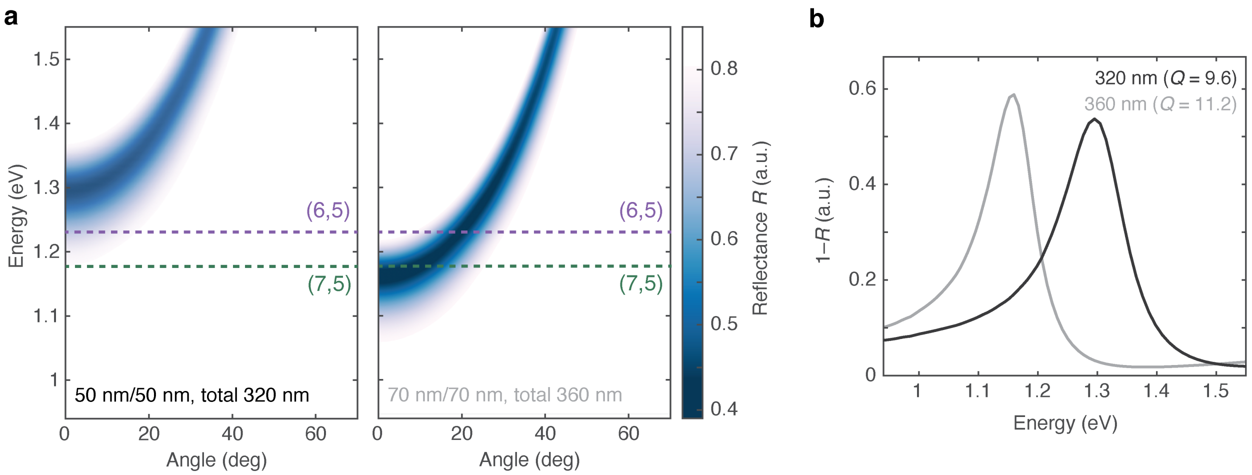

Supplementary Figure 3: Simulated cavity mode energies. **a** Dispersion of an empty microcavity simulated for cases when both CNT layers 50 nm thick (left, total microcavity thickness 320 nm) and when both CNT layers 70 nm thick (right, total microcavity thickness 360 nm). The colored dashed lines indicate the S_{11} energies of the uncoupled (6,5)- and (7,5) CNTs. **b** Quality factor (Q) calculated from the plots in **a**. The black and gray traces are the $1-R$ spectrum at normal incidence obtained from **a**, for the 320 nm and 360 nm case, respectively. The Q values estimated are indicated in the figure.

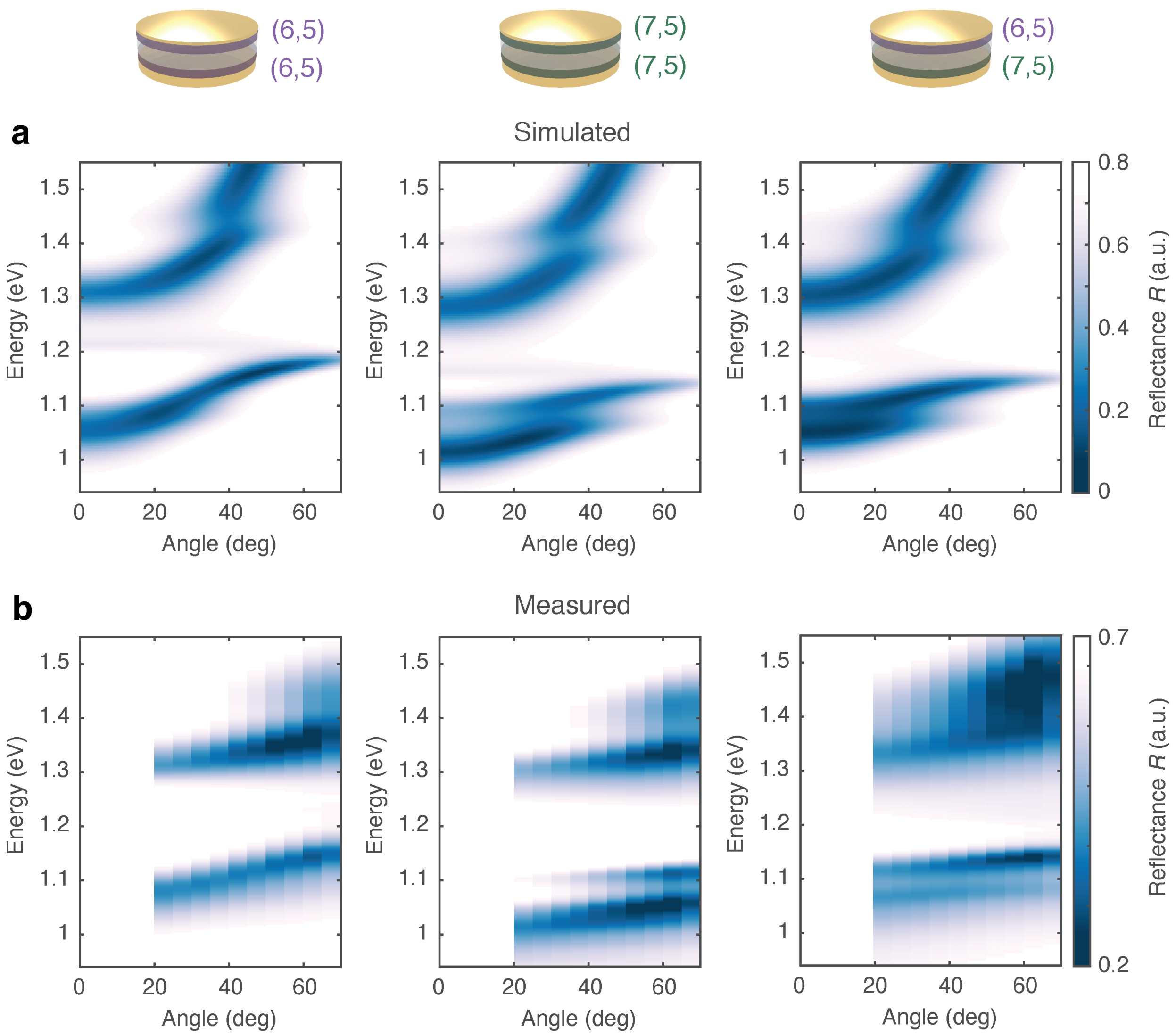

Supplementary Figure 4: Angle-dependent linear reflectance spectra of the microcavities. a Spectra simulated by the transfer matrix method for the (6,5) (left), (7,5) (middle), and mixed (6,5)/(7,5) microcavities (right). Simulations were performed for a PVA layer thickness of 150 nm and CNT layer thickness of 55 nm on both sides of the PVA layer, which yielded the best match to the experimental data shown in **b**. **b** Measured spectra of the (6,5) (left), (7,5) (middle), and mixed (6,5)/(7,5) microcavities (right). Due to the technical specification of our ellipsometer, only the 20−70° data were measured and plotted. The rightmost plot in **b** is a replicate of Fig. 1c in the main text. We note that we only use these simulations for qualitative comparison of the spectral profiles, especially towards the high-energy range, due to potential inaccuracies in modeling the background indices, potential islanding of the gold layers, etc.

2.2. Simulation of transient reflection spectra

The transient reflection spectra were simulated by scaling the optical constants to describe the bleaching of the ground-state CNT population upon excitation with the pump laser pulse. The ground-state refractive indices of both the (6,5) and (7,5) CNT films, prepared in an identical way to how they were prepared for the microcavity samples, were best-modeled with a Lorentzian/Cody-Lorentzian oscillator model with three oscillators (Supplementary Tables 1−4). The equations used and details of the Lorentzian/Cody-Lorentzian oscillator model are provided in [3].

We model the excited-state refractive indices by scaling the ground-state refractive indices as modeled above. To account for partial bleaching of the ground-state population upon excitation with the pump pulse, we introduce variable scaling factors, the "bleach terms", to each oscillator. We obtain the excited-state reflectance spectra using the transfer matrix method with these scaled refractive indices as input. The difference calculated by subtracting the ground-state ("pump off") spectra from the excited-state ("pump on") spectra gives the transient reflection spectra as a function of the bleach terms. We fit the simulated transient reflection spectra to the experimental ones by varying the bleach term for each oscillator. The best-fit spectra are shown in Fig. 3a−c in the main text as the darkest-shade traces. The lighter-shade traces are simulated spectra with scaled bleach terms to account for the reduction in photoexcited population at later time delays.

Supplementary Table 1: Background index (isotropic) for the (6,5) layer.

Parameter	Value
E_{inf}	0.00767
UV pole amplitude	197.6013
UV pole energy (in eV)	7.046
IR pole amplitude	0.5266

Supplementary Table 2: Oscillators for the in-plane index for the (6,5) layer.

Oscillator	Type	Amplitude	Linewidth	E_0 (eV)	E_g (eV)	E_p (eV)
1	Lorentz	0.61354	0.2548	1.106	—	—
2	Cody-Lorentz	19.333	0.0405	1.232	1.034	1.242
3	Cody-Lorentz	7.353	0.0848	1.427	1.319	1.109

Supplementary Table 3: Background index (isotropic) for the (7,5) layer.

Parameter	Value
E_{inf}	0.00014358
UV pole amplitude	205.7661
UV pole energy (in eV)	7.251
IR pole amplitude	0.4336

Supplementary Table 4: Oscillators for the in-plane index for the (7,5) layer.

Oscillator	Type	Amplitude	Linewidth	E_0 (eV)	E_g (eV)	E_p (eV)
1	Lorentz	2.071346	0.1009	1.085	—	—
2	Cody-Lorentz	30.371	0.0382	1.183	1.041	1.069
3	Cody-Lorentz	76.421	0.0569	1.376	1.320	2.249

Supplementary Note 3: Coupled oscillator model fit to the microcavity reflectance spectra

The eigenstate energies of each microcavity were modeled using a coupled oscillator model and compared with the experimental angle-dependent reflectance spectra[4, 5]. For the experimental data, the angle was controlled with $\pm 0.01^\circ$ accuracy, and the wavelength was incremented in 1 nm steps, which is well below the linewidth of all peaks. Thus, the fits were not impacted by any uncertainty in the experimental data. As shown below in Supplementary Equations (1)−(3), the (6,5)- and (7,5) microcavities were modeled with a 3×3 and 4×4 Hamiltonian, respectively, where $E_c(\theta)$ is the cavity mode energy as a function of angle θ ($E_c(\theta) = E_c(0)/\sqrt{1-(sin\theta/n_{\text{eff}})^2}$, n_{eff} is the effective refractive index), $E_{6,5}$, $E_{7,5}$, and $E_{7,6}$ are the S_{11} energies of the (6,5), (7,5), and (7,6) CNTs, and $E_{6,5/\text{PSB}}$ and $E_{7,5/\text{PSB}}$ are the phonon sideband energies of the (6,5) and (7,5) CNTs, respectively (Fig. 1b in the main text). The mixed (6,5)/(7,5) microcavity was modeled with a 6×6 Hamiltonian by including all five exciton energies above. The off-diagonal elements g_i indicate the strength of light-matter coupling.

(6,5) microcavity:

$$\begin{pmatrix} E_c(\theta) & g_1 & g_2 \\ g_1 & E_{6,5} & 0 \\ g_2 & 0 & E_{6,5/\text{PSB}} \end{pmatrix} \begin{pmatrix} \alpha_1 \\ \alpha_2 \\ \alpha_3 \end{pmatrix} = E \begin{pmatrix} \alpha_1 \\ \alpha_2 \\ \alpha_3 \end{pmatrix} \tag{1}$$

(7,5) microcavity:

$$\begin{pmatrix} E_c(\theta) & g_1 & g_2 & g_3 \\ g_1 & E_{7,5} & 0 & 0 \\ g_2 & 0 & E_{7,5/\text{PSB}} & 0 \\ g_3 & 0 & 0 & E_{7,6} \end{pmatrix} \begin{pmatrix} \alpha_1 \\ \alpha_2 \\ \alpha_3 \\ \alpha_4 \end{pmatrix} = E \begin{pmatrix} \alpha_1 \\ \alpha_2 \\ \alpha_3 \\ \alpha_4 \end{pmatrix} \tag{2}$$

Mixed (6,5)/(7,5) microcavity:

$$\begin{pmatrix} E_c(\theta) & g_1 & g_2 & g_3 & g_4 & g_5 \\ g_1 & E_{6,5} & 0 & 0 & 0 & 0 \\ g_2 & 0 & E_{6,5/\text{PSB}} & 0 & 0 & 0 \\ g_3 & 0 & 0 & E_{7,5} & 0 & 0 \\ g_4 & 0 & 0 & 0 & E_{7,5/\text{PSB}} & 0 \\ g_5 & 0 & 0 & 0 & 0 & E_{7,6} \end{pmatrix} \begin{pmatrix} \alpha_1 \\ \alpha_2 \\ \alpha_3 \\ \alpha_4 \\ \alpha_5 \\ \alpha_6 \end{pmatrix} = E \begin{pmatrix} \alpha_1 \\ \alpha_2 \\ \alpha_3 \\ \alpha_4 \\ \alpha_5 \\ \alpha_6 \end{pmatrix} \tag{3}$$

The diagonalized matrix, E, on the right-hand side contains the eigenstate energies in each polariton system with Hopfield coefficients $|\alpha_i|^2$ that describe the contribution of the constituent exciton and photon

states. The peak positions identified in measured angle-dependent linear reflectance spectra (Supplementary Fig. 4b) were globally fit at all angles to the eigenstate energies from the models above with $E_c(0)$, n_{eff}, and g as variable parameters. Only the coupling terms for $E_{6,5}$ and $E_{7,5}$, *i.e.*, g_1 in the single band-gap microcavities and g_1, g_3 in the mixed (6,5)/(7,5) sample were varied independently. The other coupling terms were scaled by the relative transition dipole strength, *i.e.*, square root of the relative peak intensity, given that the coupling strength is proportional to the transition dipole strength. The model yields good agreement with the measured spectra, as shown in Supplementary Fig. 5 and Fig. 1c in the main text. For the mixed (6,5)/(7,5) microcavity, the eigenstate at $\sim$1.22 eV is not visible in the contour plot shown in Fig. 1c in the main text due to its weak intensity, but a second-derivative analysis of the spectra reveals a local minimum at this eigenstate energy (Supplementary Fig. 6). There is no anti-crossing between the photon dispersion and the (7,5) S_{11} exciton peak, because our system has multiple exciton modes coupling to the cavity mode, rather than the more conventional system with a single exciton mode, as has been reported previously [6].

We note that the coupled oscillator model presented here, although extensively used to describe and model polariton spectra in the literature, is a simplified version of the extended Hamiltonian used for Redfield calculations later in the manuscript, and so we use the extended Hamiltonian to interpret and discuss our data in all other sections of the manuscript to prevent confusion.

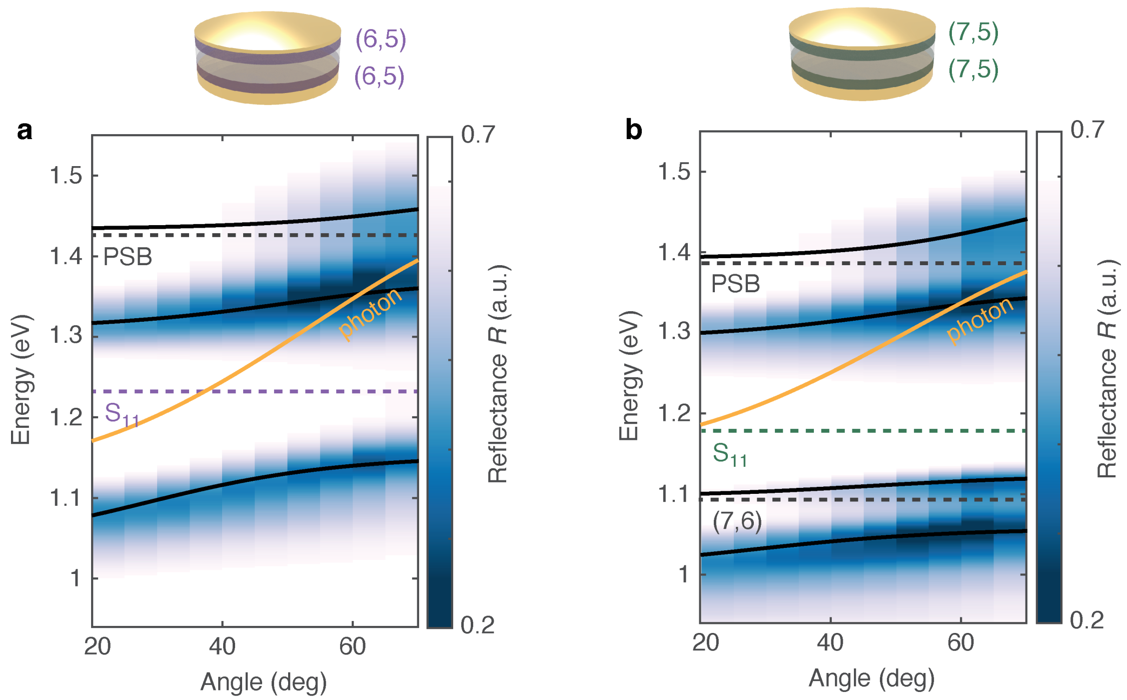

Supplementary Figure 5: Angle-dependent reflectance spectra and coupled oscillator model fits of the single band-gap microcavities. The contour plots show the measured reflectance spectra for the (6,5) (**a**) and (7,5) (**b**) microcavities. Black solid lines plot the coupled oscillator model fits to the experimental spectra, and the yellow solid lines are the photon dispersion profiles determined from the model. Dashed lines indicate the uncoupled exciton energies as labeled.

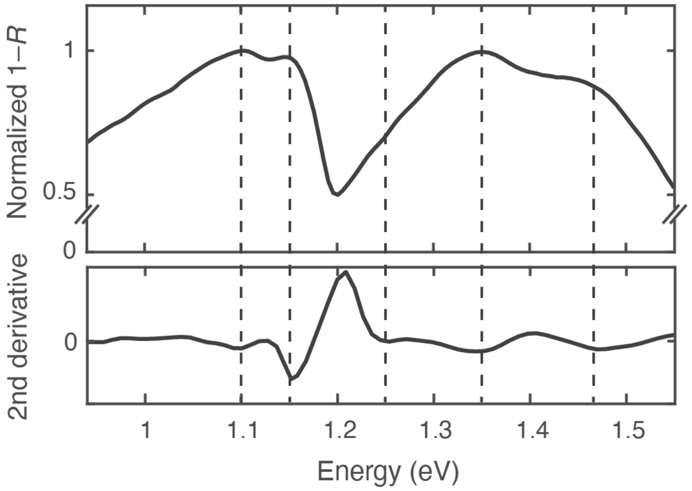

Supplementary Figure 6: Second-derivative analysis of the mixed (6,5)/(7,5) microcavity reflectance spectra. Top panel shows the $1-R$ spectrum measured at 40° angle. Bottom panel shows the second derivative of the top panel, where the peak positions are revealed as local minima (indicated with dashed lines).

Supplementary Note 4: Supplementary data from the cavity-less control sample.

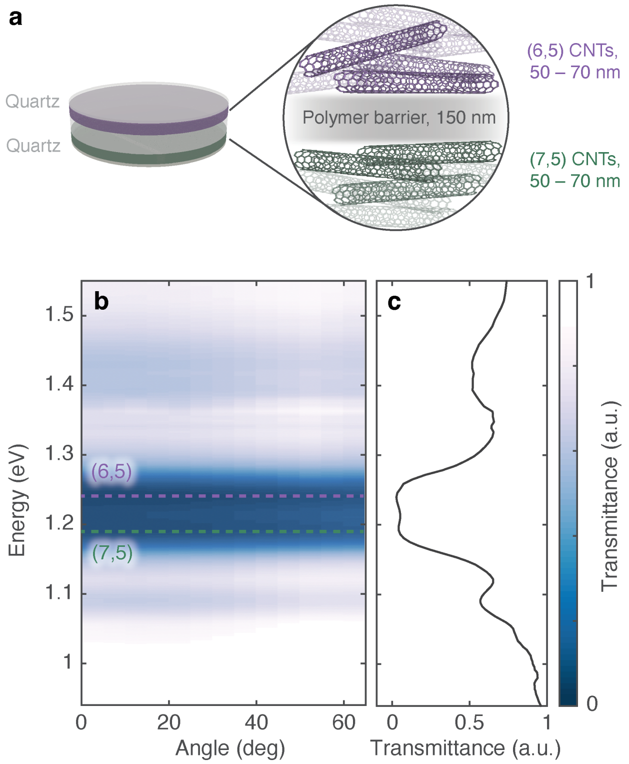

Supplementary Figure 7: Characterization of the cavity-less control sample. a Schematic illustration of the cavity-less control sample, where the CNT and PVA layers are embedded within a pair of glass substrates. **b** Angle-dependent transmittance spectra. The dashed lines label the S_{11} energies of the (6,5)- and (7,5) CNTs. **c** Slice taken at normal incidence from **b**.

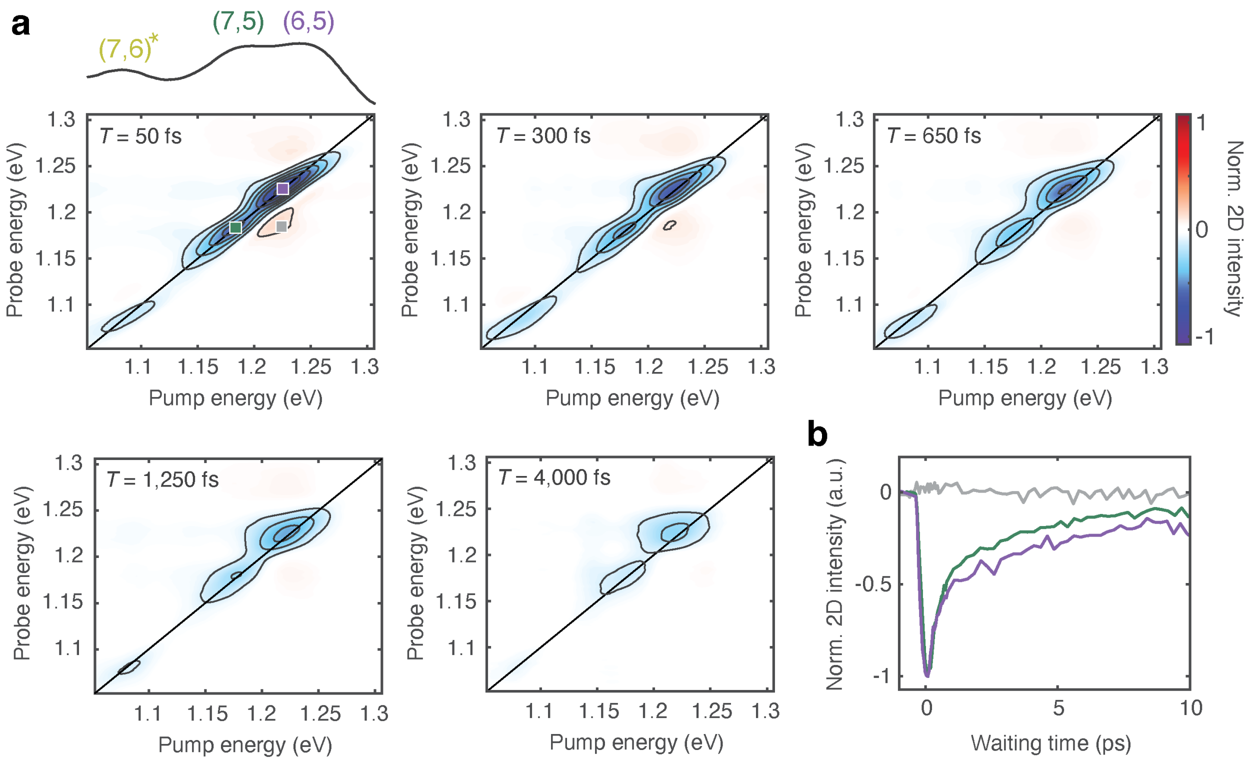

Supplementary Figure 8: 2DWL spectra of the cavity-less control sample. a 2DWL spectra of the cavity-less control (see Supplementary Fig. 7) measured at waiting times as indicated in each spectrum. The linear absorption of this sample within the spectral range of our 2DWL measurement is shown above the $T = 50$ fs spectrum. All spectra are normalized to the maximum and minimum 2D intensity of the $T = 50$ fs spectrum. **b** Waiting time traces of the (6,5)- (purple) and (7,5) diagonal peaks (green) as well as the intersection of the (6,5) pump and (7,5) probe energies (gray), as also labeled with squares in the $T = 50$ fs spectrum in **a**. All traces are normalized to the maximum magnitude of the (6,5) diagonal peak.

Supplementary Note 5: Redfield theory

5.1 Extended description for the calculation of linear spectrum and population dynamics

We calculate both the $1-R$ (absorption) spectrum and kinetics from the Hamiltonian, given in Equation (1) the main text:

$$\hat{\mathscr{H}} = \sum_i \hbar\omega_i|i\rangle\langle i| + \sum_i\sum_j J|i\rangle\langle j| + \hbar\omega_c a^\dagger a + \sum_i g_i(|G\rangle\langle i|a^\dagger + |i\rangle\langle G|a) \quad (4)$$

where ω_i is the transition frequency of the ith chromophore, J is the nearest-neighbor inter-tube coupling, ω_c is the cavity resonance frequency, $a^\dagger$ and a are photon creation and annihilation operators, and g_i is the light-matter coupling strength to each CNT. The collective coupling strength g_{tot} is given by:

$$g_{tot} = \sum_k \mu_k \sqrt{\frac{N_k \hbar\omega_c}{2V\varepsilon_o}} \quad (5)$$

where μ_k and N_k are the transition dipole and number of the chromophores of that type (k = (6,5) or (7,5)), respectively, and V is the mode volume. We fix the S_{11} energies of the (6,5) ($\omega_{(6,5)}$) and (7,5) nanotube states ($\omega_{(7,5)}$) to be 1.233 eV and 1.181 eV, respectively, to reproduce experimentally measured absorption maxima (see Fig. 1b in the main text). The CNTs are modeled as hexagonally packed bundles of 36 nanotubes. We set $J = -10$ meV, only among the same band-gap CNTs, which is consistent with a ~ 10 meV redshift in the absorption peak in a film of (6,5) CNTs relative to isolated CNTs in solution [7]. This coupling value and packing structure also reproduce the (6,5)-to-(7,5) energy transfer times previously measured in mixed CNT bundles, as discussed below in Supplementary Note 5.2 [8]. One collective light-matter coupling strength ($g = g_{tot}$) is chosen between the cavity photon and the S_{11} state of the (6,5) CNTs, and g was scaled to 75% for the (7,5) nanotubes to reflect their smaller oscillator strength [9, 10].

We diagonalize the Hamiltonian in Supplementary Equation (4) to get the polariton eigenstate energies and their transition dipoles, which we then take to the fourth power, convolve with a Gaussian lineshape (with a single linewidth of 30 meV for all eigenstates), and compare to a diagonal slice through an experimental 2DWL spectrum. We determine the cavity mode energy (ω_c) and light-matter coupling strength (g) that best describes our experimental data by fitting the simulated spectrum from above to the experimental diagonal slice at $T = 100$ fs (see Fig. 4c in the main text). As discussed in the main text, the best fit occurs when $\omega_c = 1.217$ eV and $g = 42.9$ meV. Note that this cavity mode energy is negatively detuned from the (6,5) S_{11} state, but positively detuned from the (7,5) S_{11} state.

The dynamics of the system are modeled using Redfield theory under the secular approximation, which assumes weak coupling of the system to a heat bath of harmonic oscillators [11–13]. The Redfield equation (Supplementary Equation (6)) is a second-order quantum master equation that describes the evolution of the

reduced density matrix, ρ:

$$\frac{d}{dt}\rho_{a,b} = -(\omega_a - \omega_b)\rho_{a,b} + \sum_{c,d} R_{a,b,c,d}\rho_{c,d} \tag{6}$$

$R_{a,b,c,d}$ is the Redfield tensor and is calculated from the following:

$$R_{a,b,c,d} = \Gamma^{(+)}_{d,b,a,c} + \Gamma^{(-)}_{d,b,a,c} - \delta_{d,b}\sum_e \Gamma^{(+)}_{a,e,e,c} - \delta_{a,c}\sum_e \Gamma^{(+)}_{d,e,e,b} \tag{7}$$

with

$$\Gamma^{(+)}_{a,b,c,d} = \int_0^\infty e^{-i(\omega_c-\omega_d)\tau} \sum_i C(\tau) K^{(i)}_{a,b} K^{(i)}_{c,d} d\tau \tag{8}$$

and

$$\Gamma^{(-)}_{a,b,c,d} = \int_0^\infty e^{-i(\omega_c-\omega_d)\tau} \sum_i C^*(\tau) K^{(i)}_{a,b} K^{(i)}_{c,d} d\tau \tag{9}$$

where $K^{(i)}_{a,b} = \langle a|i\rangle \langle i|b\rangle$ is the system-bath coupling operator and $C(\tau)$ is the thermal correlation function, expressed in terms of a spectral density, $\mathscr{J}(\omega)$:

$$C(\tau) = \frac{1}{\pi}\int_0^\infty \mathscr{J}(\omega)(\coth(\frac{\omega}{2k_BT})\cos(\omega\tau) - i\sin(\omega\tau))d\omega \tag{10}$$

The functional form of the spectral density and its impact on the population dynamics are discussed below in Supplementary Note 5.3.

The transition rate between states is given by

$$k_{ab} \sim \mathscr{J}(\omega_{ab}) \sum_i |\langle a|i\rangle\langle i|b\rangle|^2 \tag{11}$$

where $\mathscr{J}$ is the spectral density of the bath, a and b are eigenstates, and i is a state in the site basis. Thus, the rates are set by the overlap in the site basis and the strength of the bath coupling. For the bath coupling, we used a spectral density based on the resonance Raman spectrum of CNT bundles in solution, which contains one peak at 40 meV for the radial breathing mode and another peak at 200 meV for the G-band [14, 15]. We have also used the non-resonance Raman spectrum and have included low frequency ($<$ 20 meV) librations, which each produce trivial changes (Supplementary Note 5.3 and Supplementary Fig. 11).

We phenomenologically include the lifetimes of the polariton states in the Redfield equation:

$$\frac{d}{dt}\rho_{aa} = -(\frac{c^{cav}_a}{T_{cav}} + \frac{c^{mol}_a}{T_{mol}})\rho_{aa} + \sum_b R_{a,a,b,b}\rho_{bb} \tag{12}$$

where c^{cav}_a and c^{mol}_a are the cavity and molecular compositions of eigenstate a, and T_{cav} and T_{mol} are the lifetimes of the cavity and molecular basis states. We set the cavity lifetime to 10 fs because of the low

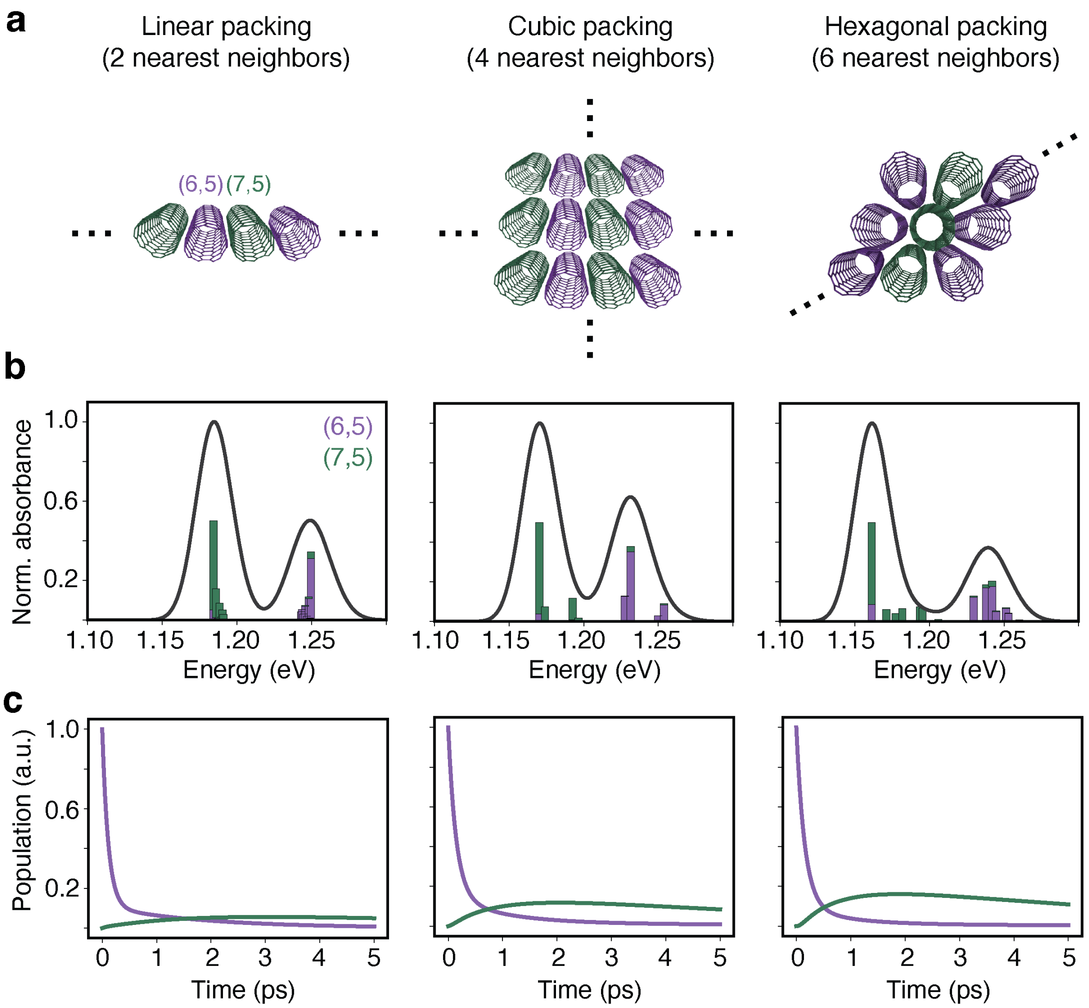

Supplementary Figure 9: Packing structure and simulation of mixed CNT bundle dynamics. a Different packing structures of the mixed CNT bundles as described in the text. **b** and **c** show the simulated linear absorption spectra (**b**, black line; colored vertical bars illustrate the transition dipoles) and (6,5)-to-(7,5) energy transfer dynamics (**c**) for each packing structure shown in **a**.

quality factor of metallic mirror cavities (Supplementary Fig. 3b). We set the molecular lifetime to 10 ps, which we know from prior work on CNT films [7, 8, 16]. According to Supplementary Equation (12), less than 10% photon character is needed to have a lifetime longer than 100 fs. This approach is similar to that used by Michetti and La Rocca [17] to model the photoluminescence spectra of organic exciton-polaritons.

5.2 Modeling of dynamics for mixed CNT bundles

To validate our choice of inter-tube coupling (J) and CNT energies ($\omega_{(6,5)}$ and $\omega_{(7,5)}$), we simulate the population dynamics of a mixed bundle of (6,5) and (7,5) CNTs, the energy transfer dynamics of which

were previously measured with 2DWL [8]. The Hamiltonian of this cavity-free system is given by:

$$\mathscr{H}^{bundle} = \sum_i \hbar\omega_i |i\rangle\langle i| + \sum_i \sum_j J|i\rangle\langle j| \tag{13}$$

We consider three different CNT packing structures: linear (two nearest neighbors), cubic (four nearest neighbors), and hexagonal (six nearest neighbors), as shown in Supplementary Fig. 9a. We find that the choice of packing does not drastically affect the spectra nor the dynamics (Supplementary Fig. 9b, c).

Using a modest system-bath coupling strength (peak value of the spectral density) of 20 meV, we are able to reproduce the $\sim$1 ps (6,5)-to-(7,5) energy transfer timescale reported in Flach *et al.* [8]. The physical picture underlying our model is that the bath, made up of the phonons of the CNT bundles, is able to stimulate transitions between the eigenstates of the bundle when the difference in energy between the eigenstates matches the frequency of the bath mode(s). These simulations demonstrate the robustness of our model to different packing structures of CNTs.

5.3 Dynamics within the energy cascade

Electronic coupling creates a manifold of bright states in between the UP and LP bands. Upon photoexcitation into the UP, energy slowly cascades down this manifold of states until it finally reaches the short-lived LP state, upon which the excitation returns to the ground state. Supplementary Fig. 10a,b shows additional kinetic traces where the energy of the initial or final state was varied, and Supplementary Fig. 10c plots the lifetime of several states within the manifold.

5.4 Effect of bath spectral density on population dynamics

Spectral density describes the coupling of the bath at each frequency to the eigenstates of the system [11]. While the spectral density can be described as a sum over individual oscillators with distinct frequencies and couplings (Supplementary Equation (14)), a functional form for the spectral density is usually chosen, such as the Debye form, given in Supplementary Equation (15):

$$\mathscr{J}(\omega) = \sum_k \frac{c_k^2}{\omega_k} \delta(\omega - \omega_k) \tag{14}$$

$$\mathscr{J}^{Debye}(\omega) = 2\lambda\Omega \frac{\omega}{\omega^2 + \Omega^2} \tag{15}$$

where Ω is a characteristic frequency and λ is the reorganization energy. The Debye form is typically assumed to describe the dense vibrational landscape of organic chromophores [12, 13]. Due to the high symmetry of CNTs, the number of phonon modes that couple to the excitons in CNTs is greatly reduced [14, 15, 18]. To

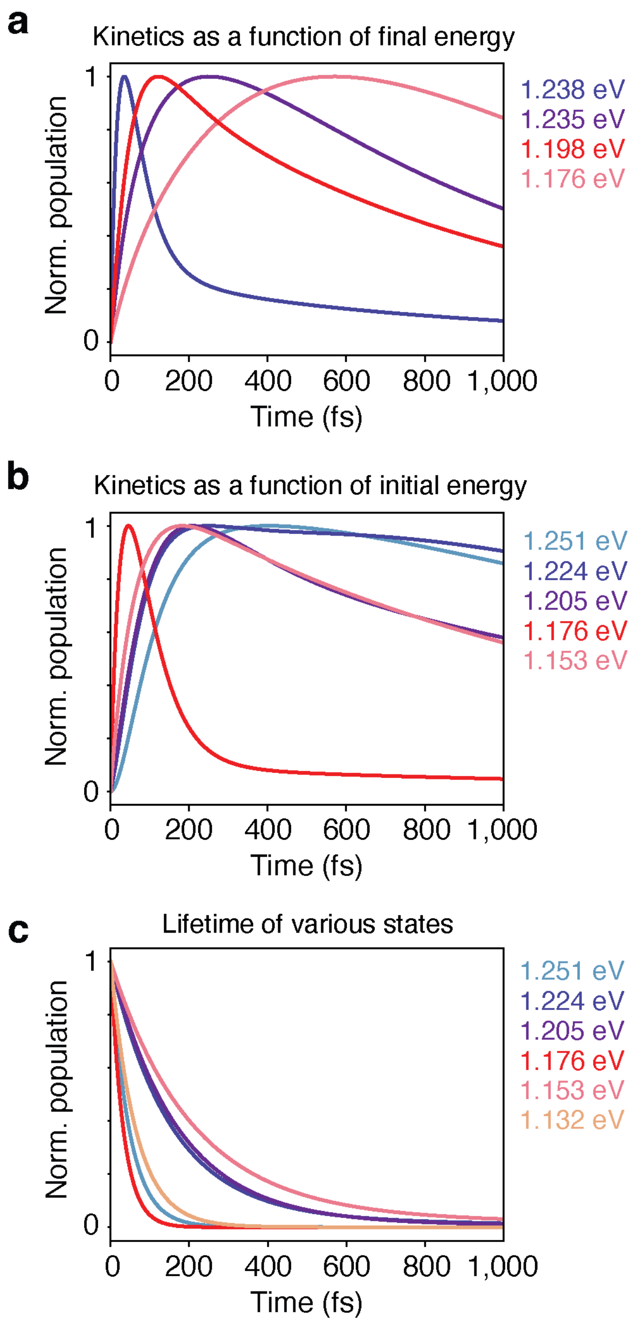

Supplementary Figure 10: Additional dynamics traces within the manifold. a,**b** Additional traces of energy relaxation in the mixed (6,5)/(7,5) microcavity. **a** shows the kinetics as a function of the energy of the final state upon excitation of the UP, at 1.251 eV. **b** shows the kinetics of relaxation to the LP, at 1.132 eV, as a function of the energy of the initial state. **c** Lifetime of various states within the manifold with energy levels as color-coded and indicated in the legend.

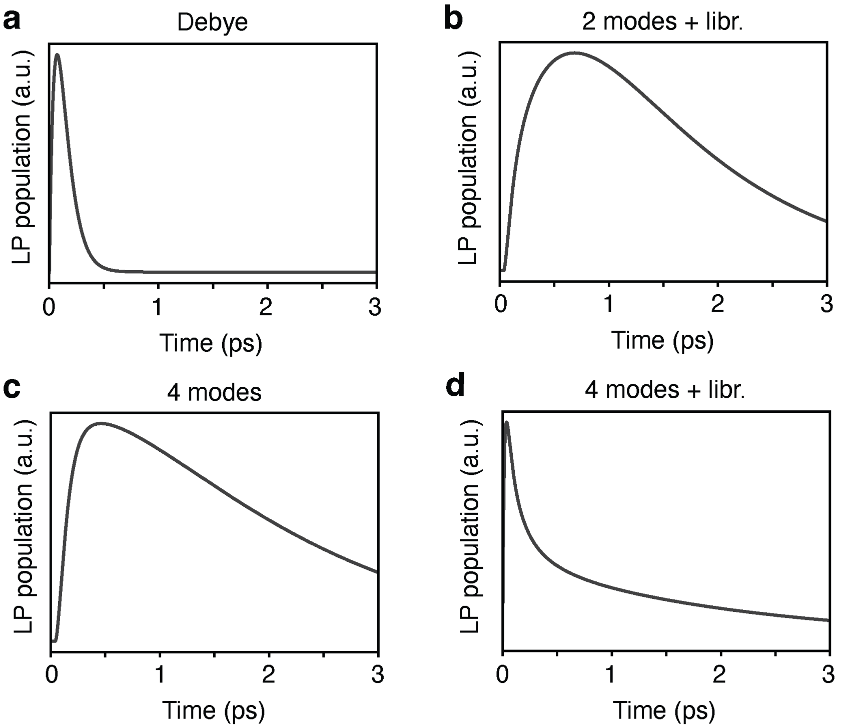

Supplementary Figure 11: Dependence of LP dynamics on spectral density. Population dynamics of the LP band upon photoexcitation into the UP band calculated using the spectral densities as labeled on top of each panel.

describe this sparse vibrational landscape, we use the following functional forms:

$$\mathscr{J}^{2mode}(\omega) = \lambda\left(\frac{1}{1+(\frac{\omega-200\text{ meV}}{5/2\text{ meV}})^2} + \frac{0.2}{1+(\frac{\omega-40\text{ meV}}{20/2\text{ meV}})^2}\right) \quad (16)$$

which describes the G-band and radial breathing mode of CNTs, and an extended form

$$\mathscr{J}^{4mode}(\omega) = \mathscr{J}^{2mode} + \lambda\left(\frac{0.6}{1+(\frac{\omega-330\text{ meV}}{5/2\text{ meV}})^2} + \frac{0.1}{1+(\frac{\omega-160\text{ meV}}{5/2\text{ meV}})^2}\right) \quad (17)$$

where the two new terms describe the G'-band and the D-band. We also consider a term to describe extremely low-frequency librational modes:

$$\mathscr{J}^{lib}(\omega) = \lambda(0.2e^{\frac{-\omega}{20\text{meV}}}) \quad (18)$$

Using different combinations of these bath modes, we calculate the population dynamics of the mixed microcavity. Shown in Supplementary Fig. 11a are the dynamics of the LP state calculated using a Debye spectral density. Despite the presence of inter-tube coupling, rapid transfer to the LP state is observed, followed by a rapid decay, similarly to the cases presented in Fig. 5d, f in the main text. The rapid dynamics of the LP state are due to an enhancement of the direct UP-to-LP energy transfer pathway over the cascading

energy transfer pathway. The enhancement comes from using a broad spectral density that has bath modes coupled to the system at the Rabi splitting energy. Supplementary Fig. 11b−d show LP dynamics calculated from $\mathscr{J}^{2mode}$ or $\mathscr{J}^{4mode}$, with and without librational modes (the data shown and discussed in the main text are calculated with the $\mathscr{J}^{2mode}$ functional form), all of which are more discretized than the broad Debye form. These discretized spectral densities do not have bath modes that couple at the Rabi splitting energy, which has the effect of suppressing direct UP-to-LP transfer. After excitation of the UP band, energy transfer is only efficient to other bright and dark states that are $\sim$40 meV away from the UP band, or closer if $\mathscr{J}^{lib}$ is included. Following this initial energy transfer step, a series of $<$ 40 meV hops down the manifold of bright and dark molecular states takes place, eventually reaching the LP state. As stated in the main text, the LP state has a short lifetime, but there is a long-lived population in the LP state because of the cascading from other molecular states. We interpret the decay of the UP-LP cross peak in the 2DWL spectra as the time it takes to cascade all the way through the manifold of molecular states. Because the G-band, G'-band, and D-band are all higher in energy than the Rabi splitting energy, they do not contribute appreciably to the population dynamics. With stronger light-matter coupling, it is possible that these modes could enhance direct UP-to-LP transfer.

5.5 Brightening of dark molecular states

As discussed in the main text, intermolecular coupling leads to the creation of bright molecular states, *i.e.*, states with low photon content, that influence the dynamics of the polaritonic system. To understand why some molecular states gain oscillator strength, we plot the eigenvector coefficients in Supplementary Fig. 12. These eigenvector coefficients mix the transition dipole moments of all the sites and determine whether the eigenstate is bright or dark.

The eigenvectors for the UP and LP bands are shown in Supplementary Fig. 12a, b. Almost all of the molecular coefficients are in phase with each other, meaning that these transition dipoles add to create a large overall transition dipole moment. Coefficients are shown in Supplementary Fig. 12c, d for two different dark states, where the coefficients of the molecular states are out of phase with each other, thereby making the overall transition dipole moment zero. Some of the molecular states become bright because the symmetry of the eigenvectors is broken by the intermolecular coupling. That is, the transition dipoles no longer add up to zero. Two examples are shown in Supplementary Fig. 12e, f. In the first case (Supplementary Fig. 12e), the molecular state has zero contribution from the (7,5) nanotube states and the coefficients from the (6,5) states are almost out of phase with each other, but not quite. This broken phase relationship leads to a non-zero transition dipole. In the second case (Supplementary Fig. 12f), the coefficients for the (6,5) nanotube states are perfectly out of phase, but there are small contributions from all of the (7,5) nanotube

states, which are all perfectly in phase, leading to a non-zero transition dipole moment. This second case highlights why having two different chromophores in the same cavity, like in our mixed cavity, enhances the presence of bright molecular states.

We note that the bright ”molecular” states, *i.e.*, states with low photon content, discussed here and ”uncoupled” states discussed in other studies [19–22] are distinct and should exhibit different behaviors. The molecular states discussed here are bright coherent states with a non-zero transition dipole moment. Uncoupled states arise due to chromophores with either zero transition dipole strength or transition dipoles not aligned with the cavity mode [19, 20].

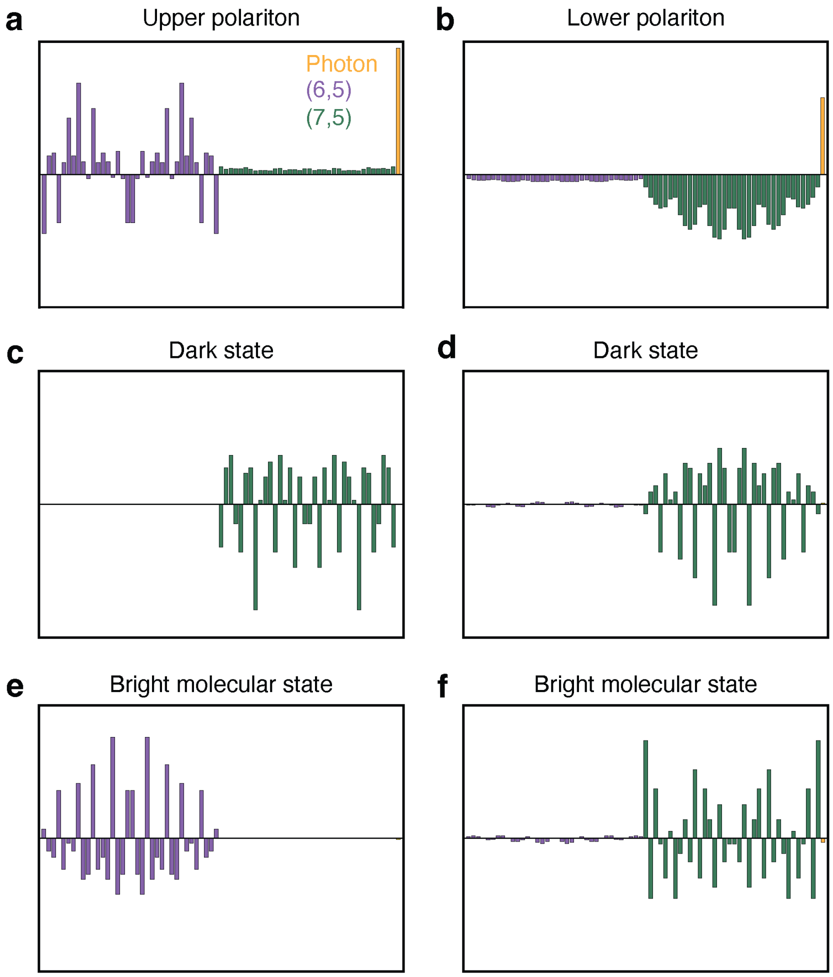

Supplementary Figure 12: Eigenvector coefficients. Coefficients of several eigenstates shown as bar plots with color codes as displayed in the legend.

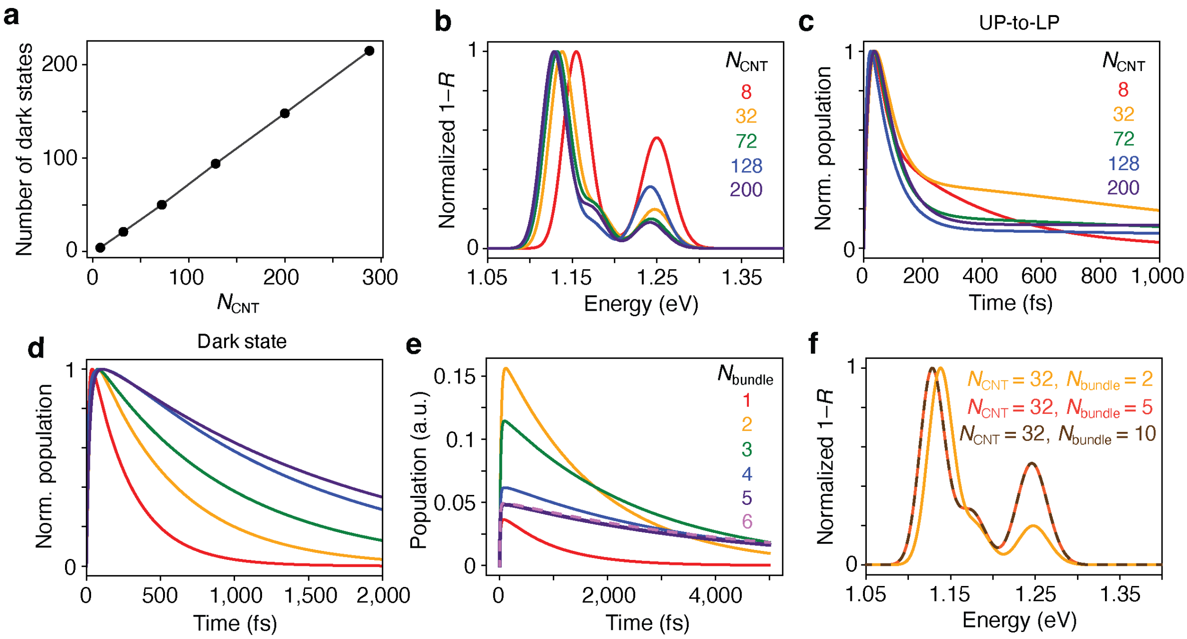

Supplementary Figure 13: Impact of the system size on the simulated spectra and kinetics. a Number of dark states as a function of system size N_{CNT}, which denotes the number of CNTs per bundle. **b**−**d** Simulated $1-R$ spectra (**b**) and kinetics (UP-to-LP, **c**; dark state, **d**) as N_{CNT} is increased from 8 to 200 as shown in the legend ($N_{\mathrm{bundle}} = 2$, one of which is a bundle of (6,5) and the other is of (7,5)). **e** Plot of the sum of all dark state population over time upon excitation into the UP band as a function of the number of bundles (N_{bundle}) when $N_{\mathrm{CNT}} = 36$. The traces converge at $N_{\mathrm{bundle}} = 5$. **f** Simulated $1-R$ spectra for $N_{\mathrm{CNT}} = 32$ at three different bundle sizes.

5.6 System size dependence of the spectra and dynamics

In order to test if our findings are a result of simulating systems with a small number of dark states, we explored the impact of the system size by changing the size of the Hamiltonian. We explore the impact of both the number of CNTs in each bundle, N_{CNT} (Supplementary Fig. 13a−d), and the number of bundles, N_{bundle} (Supplementary Fig. 13e,f). A constant collective coupling strength of 42.9 meV was used across varying bundle numbers and sizes, which was derived from fits to the experimental 2DWL diagonal slice (Fig. 4c in the main text). Supplementary Fig. 13a shows that the number of dark states depends linearly on N_{CNT}, as is expected, but is not $N_{\mathrm{CNT}} - 1$ as predicted by the conventional model. The remaining bright states, *i.e.*, bright polariton states as well as molecular states, depend linearly on N_{CNT} as well, indicating that they are intrinsic to the Hamiltonian and not an artifact caused by using a small system size. As shown in Supplementary Fig. 13b−d, both the spectra and dynamics converge as N_{CNT} is increased. Supplementary Fig. 13e,f illustrates that the spectra and dynamics also converge as N_{bundle} is increased.

5.7 Supplementary theory data

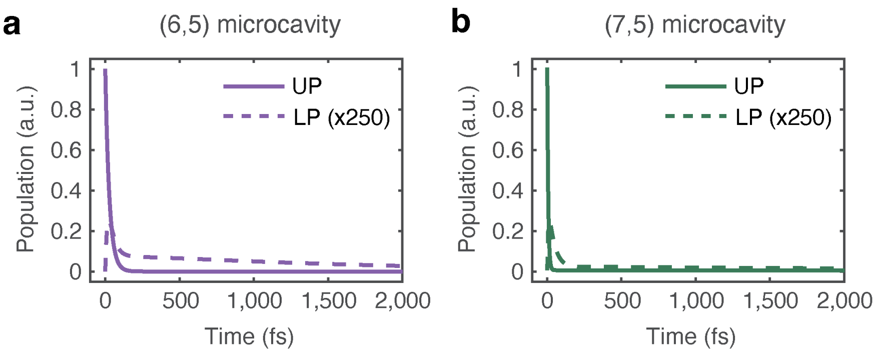

Supplementary Figure 14: Simulated UP and LP population dynamics for the single band-gap microcavities. Population dynamics of the UP (solid lines) and LP (dashed lines) for the (6,5) (**a**) and (7,5) (**b**) microcavities, calculated by Redfield theory. The traces are normalized such that the initial UP population is 1. The LP traces are plotted with a multiplication factor of 250 for clarity. Normalized LP traces are shown in Fig. 4g, h in the main text.

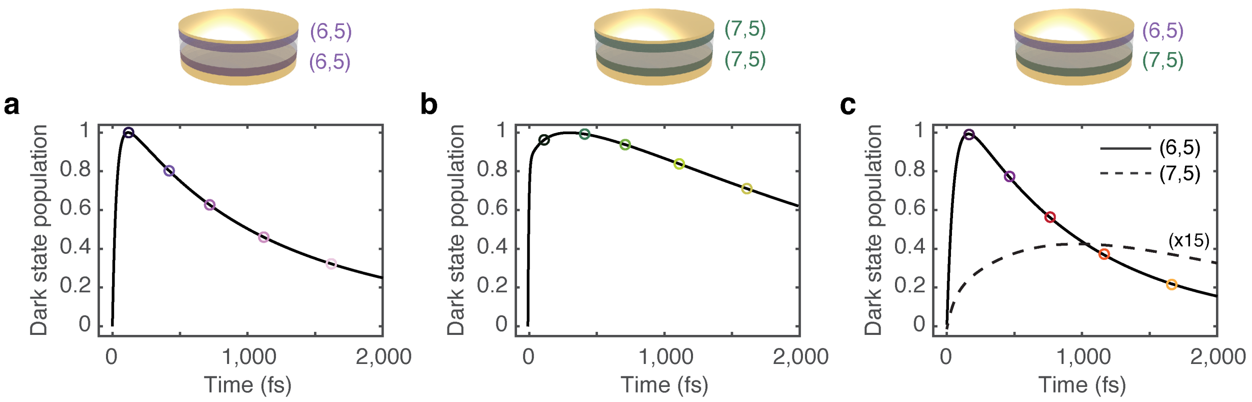

Supplementary Figure 15: Calculated dark state population of the microcavities. a–c Dark state population over time simulated by Redfield theory for the (6,5) (**a**), (7,5) (**b**), and mixed (6,5)/(7,5) microcavities (**c**). In **a**, **b**, each trace is plotted normalized to the maximum dark state population in each case. In **c**, both traces are shown normalized to the maximum dark state population of (6,5) to illustrate their different magnitudes. The normalized (7,5) trace is plotted with a multiplication factor of 15 due to its extremely small magnitude. The colored circles indicate the scaling factors for the bleach used to model the transient reflection spectra and pseudo-time traces shown in Fig. 3 in the main text.

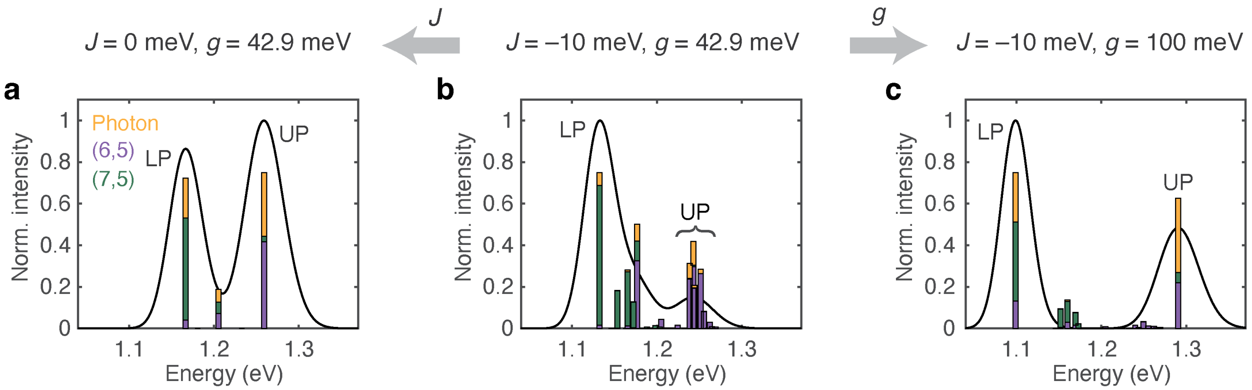

Supplementary Figure 16: Linear spectra calculated for different strengths of inter-tube and light-matter couplings. Calculated linear $1 - R$ spectra of the mixed (6,5)/(7,5) microcavity in the absence of inter-tube coupling (J) (**a**), when both J and light-matter coupling (g) are present with comparable amounts (**b**), and when g is significantly larger than J (**c**). The specific values used for each case are displayed on top. The vertical bars plot the individual eigenstates with heights proportional to the transition dipole strength. The color codes illustrate the composition of each eigenstate.

Supplementary Note 6: Supplementary experimental data

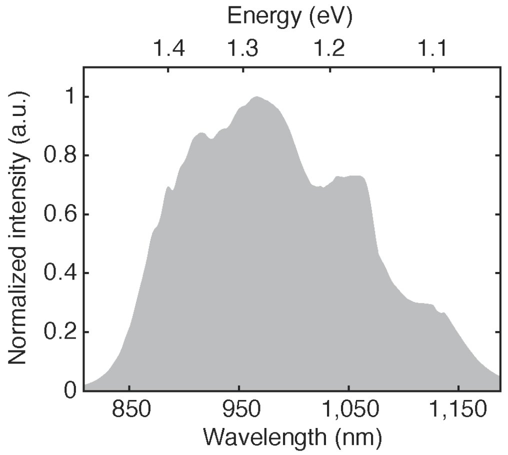

Supplementary Figure 17: White-light laser spectrum employed in the 2DWL and transient reflection measurements. The spectrum is normalized to the maximum count.

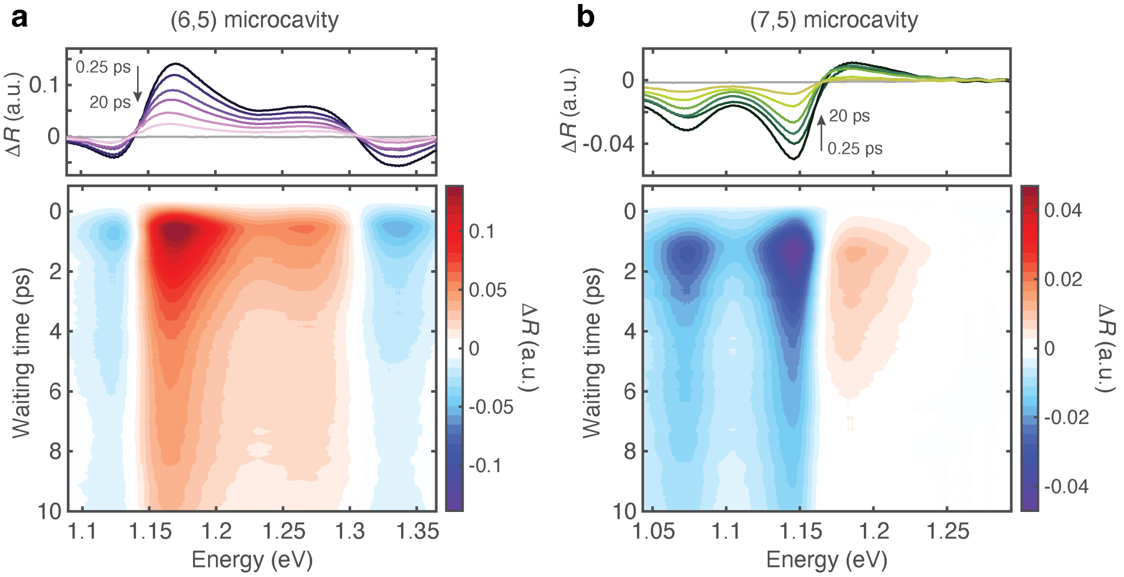

Supplementary Figure 18: Transient reflection spectra of the single band-gap microcavities. Contour maps of the transient reflection spectra of the (6,5) (**a**) and (7,5) (**b**) microcavities. Also shown are slices taken at $T = -0.5$, 0.25, 1, 2, 3, 7.5 and 20 ps on top of each contour map.

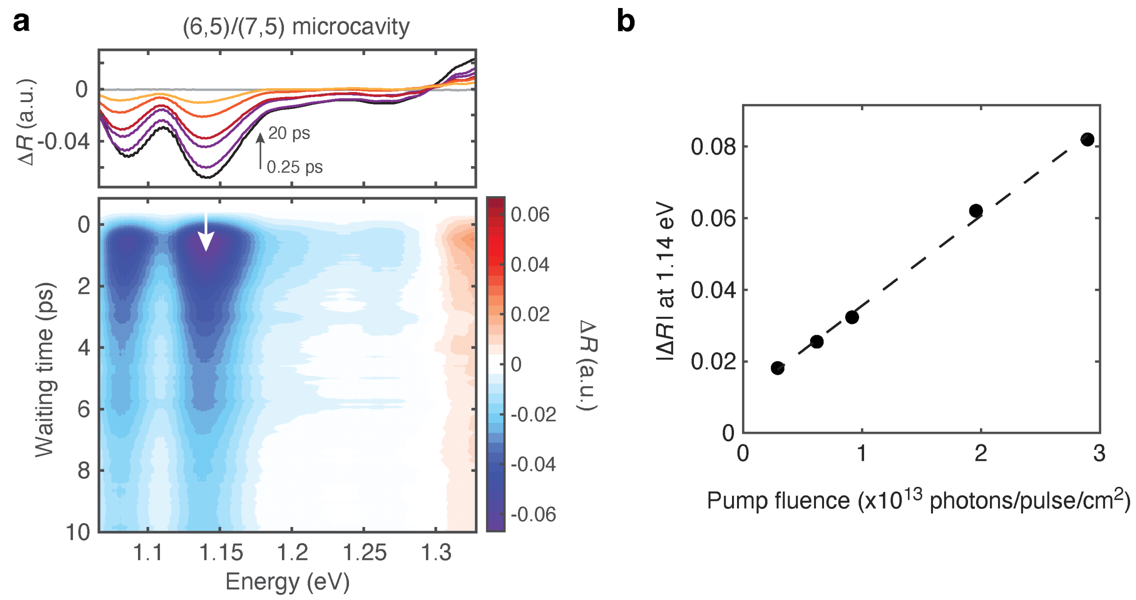

Supplementary Figure 19: Transient reflection spectra of the mixed microcavity. a Contour map of the transient reflection spectra of the (6,5)/(7,5) microcavity. Also shown are slices taken at $T = -0.5$, 0.25, 1, 2, 3, 7.5 and 20 ps on top of the contour map. **b** Magnitude of the peak at 1.14 eV (labeled with a white arrow in **a**) plotted as a function of pump fluence. The linear dependence confirms the absence of nonlinear effects in the data.

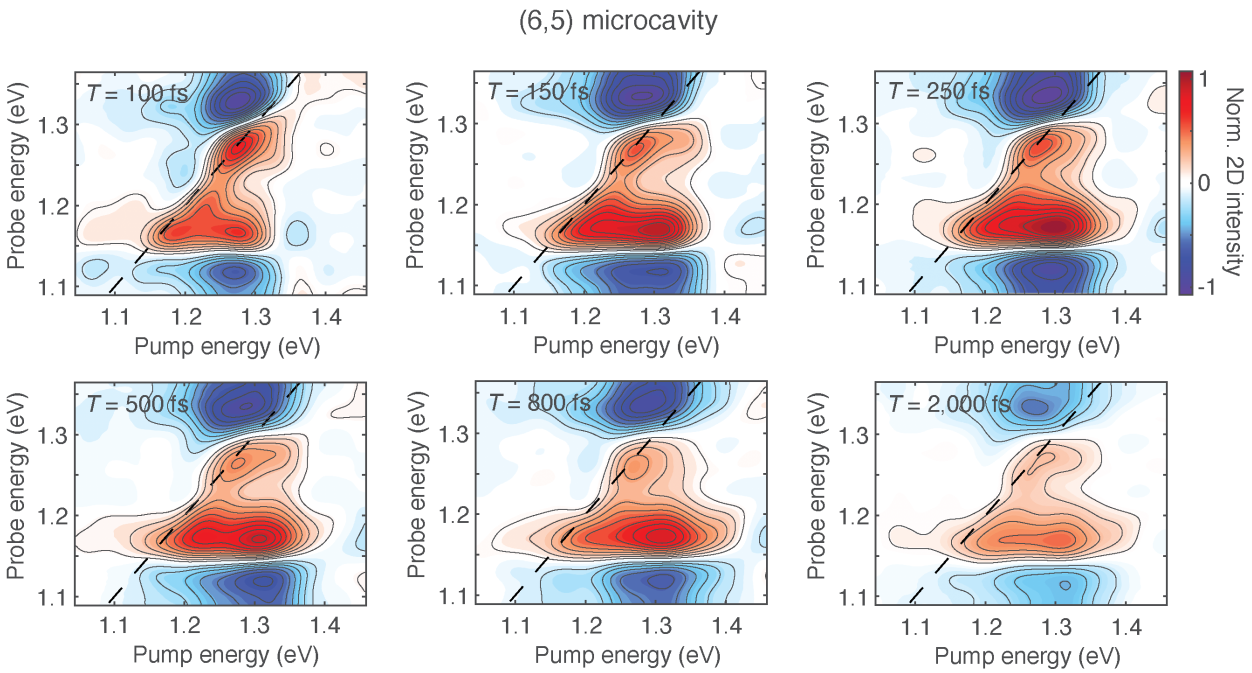

Supplementary Figure 20: Additional 2DWL spectra for the (6,5) microcavity. Waiting times are indicated in each panel. The $T = 100$ and 500 fs spectra are reproduced from Fig. 2d in the main text with slightly different probe energy axes. All spectra are normalized to the maximum magnitude of the 2D signal in the $T = 100$ fs spectrum.

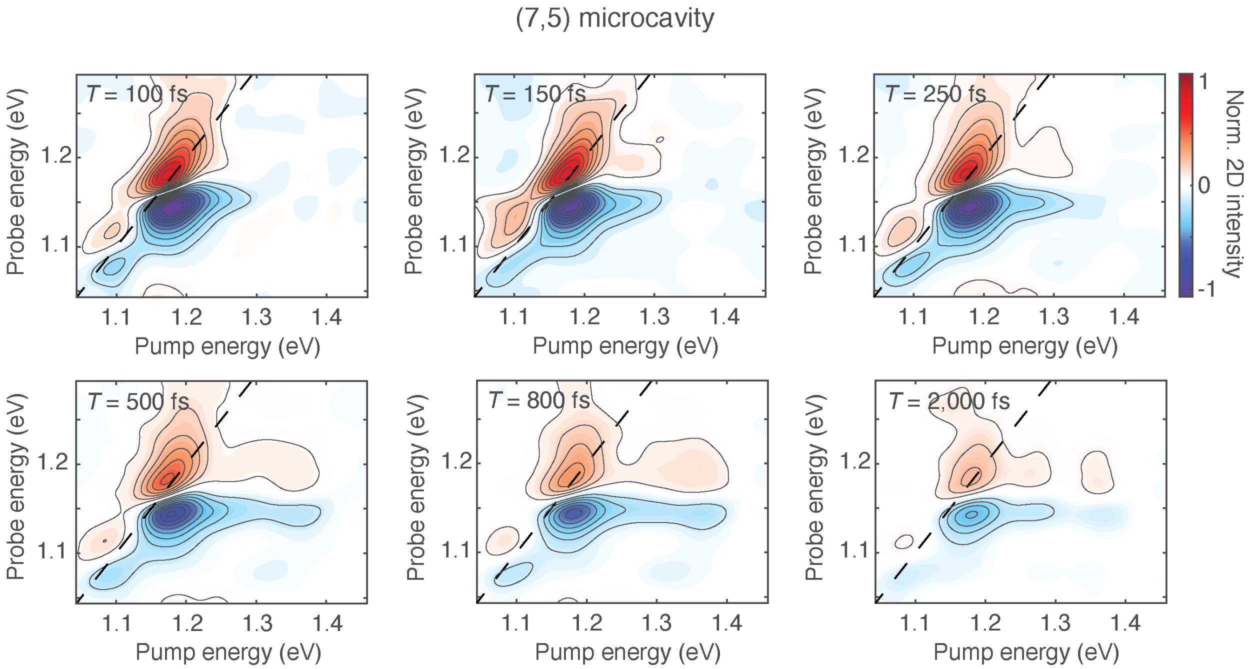

Supplementary Figure 21: Additional 2DWL spectra for the (7,5) microcavity. Waiting times are indicated in each panel. The $T = 100$ and 500 fs spectra are reproduced from Fig. 2e in the main text with slightly different probe energy axes. All spectra are normalized to the maximum magnitude of the 2D signal in the $T = 100$ fs spectrum.

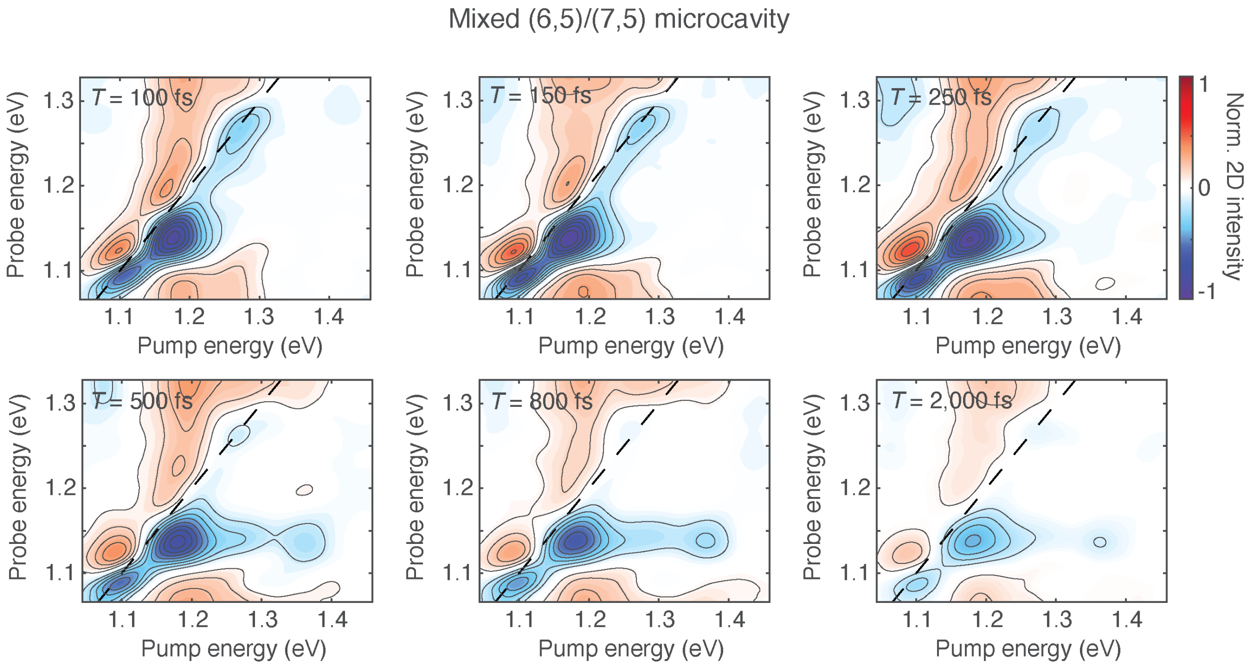

Supplementary Figure 22: Additional 2DWL spectra for the mixed (6,5)/(7,5) microcavity. Waiting times are indicated in each panel. The $T = 100$ and 500 fs spectra are reproduced from Fig. 2f in the main text with slightly different probe energy axes. All spectra are normalized to the maximum magnitude of the 2D signal in the $T = 100$ fs spectrum.

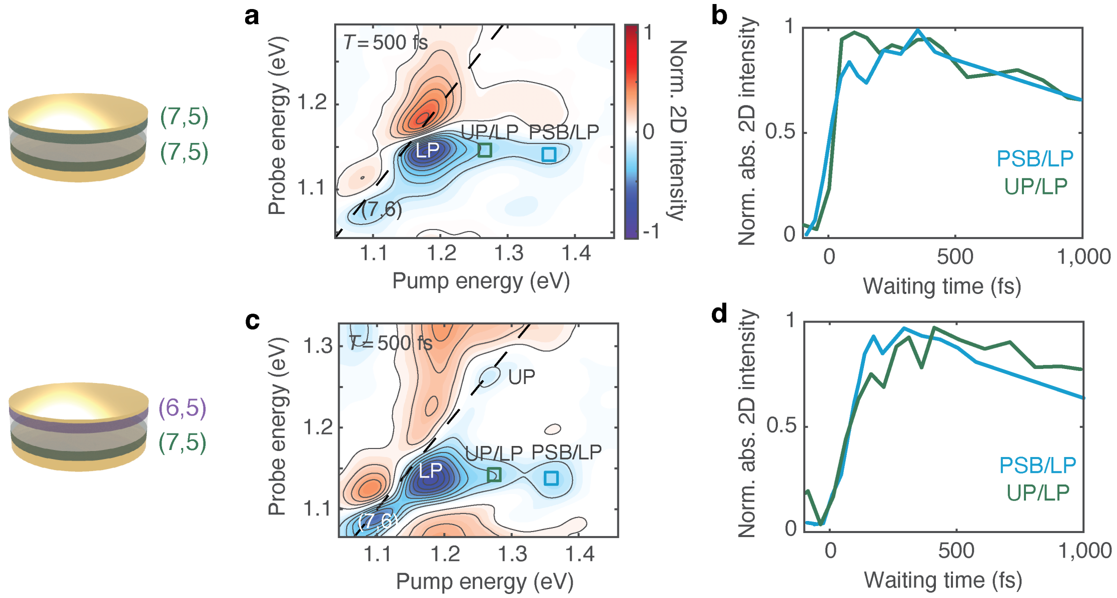

Supplementary Figure 23: Additional cross peak traces. Waiting time trace of the UP/LP (green) and PSB/LP (blue) cross peaks (labeled with colored squares in **a**, **c**, reproduced from Supplementary Figs. 21, 22) for the (7,5) (**b**) and mixed (6,5)/(7,5) (**d**) microcavities. Similarly to the case of the UP/LP cross peak, the PSB/LP shows an instantaneous growth for the (7,5) microcavity and a delayed growth for the mixed (6,5)/(7,5) microcavity.

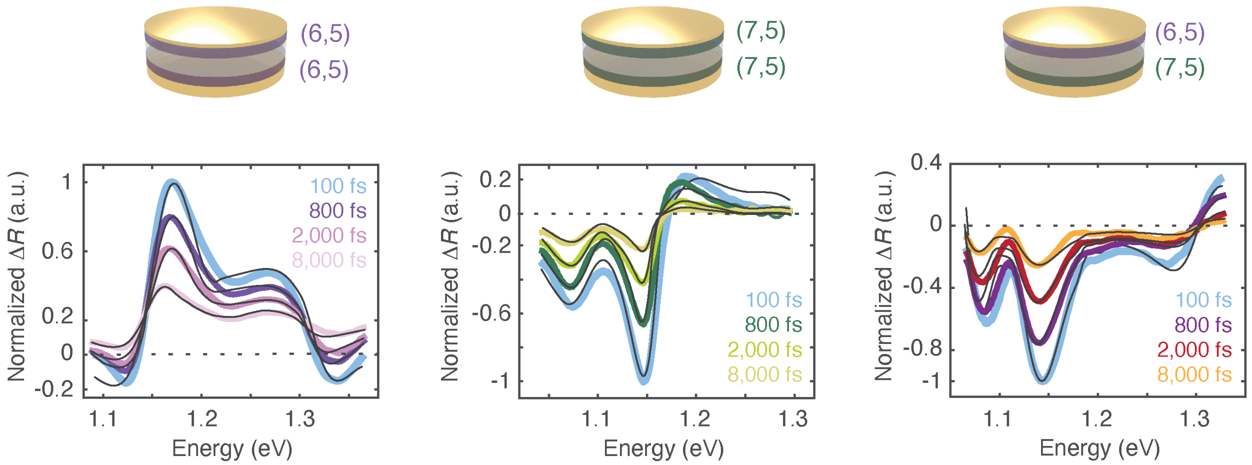

Supplementary Figure 24: Additional simulated transient reflection spectra. Overlay of measured transient reflection spectra (thick colored lines; light blue spectra are reproduced from Fig. 3a−c in the main text) and simulated spectra (black lines) at additional T points as indicated in each figure.

SUPPLEMENTARY REFERENCES

1. Coles, D. M. *et al.* Polariton-mediated energy transfer between organic dyes in a strongly coupled optical microcavity. *Nat. Mater.* **13**, 712–719 (2014).
2. Herrera, F. & Owrutsky, J. Molecular polaritons for controlling chemistry with quantum optics. *J. Chem. Phys.* **152**, 100902 (2020).
3. Woollam, J. A. *CompleteEASE Software Manual, Chapter 10* (J. A. Woollam, 2004).
4. Skolnick, M. S., Fisher, T. A. & Whittaker, D. M. Strong coupling phenomena in quantum microcavity structures. *Semicond. Sci. Technol.* **13**, 645 (1998).
5. Hertzog, M., Wang, M., Mony, J. & Börjesson, K. Strong light–matter interactions: A new direction within chemistry. *Chem. Soc. Rev.* **48**, 937–961 (2019).
6. Dhavamani, A., Haeberlé, L., Wang, J., Kéna-Cohen, S. & Arnold, M. S. Cavity-mediated hybridization of bright and dark excitons in an ultrastrongly coupled carbon nanotube microcavity. *ACS Photonics* **8**, 2375–2383 (2021).
7. Mehlenbacher, R. D. *et al.* Ultrafast exciton hopping observed in bare semiconducting carbon nanotube thin films with two-dimensional white-light spectroscopy. *J. Phys. Chem. Lett.* **7**, 2024–2031 (2016).
8. Flach, J. T., Wang, J., Arnold, M. S. & Zanni, M. T. Providing time to transfer: Longer lifetimes lead to improved energy transfer in films of semiconducting carbon nanotubes. *J. Phys. Chem. Lett.* **11**, 6016–6024 (2020).
9. Streit, J. K., Bachilo, S. M., Ghosh, S., Lin, C.-W. & Weisman, R. B. Directly measured optical absorption cross sections for structure-selected single-walled carbon nanotubes. *Nano Lett.* **14**, 1530–1536 (2014).
10. Sanchez, S. R., Bachilo, S. M., Kadria-Vili, Y., Lin, C.-W. & Weisman, R. B. (n, m)-specific absorption cross sections of single-walled carbon nanotubes measured by variance spectroscopy. *Nano Lett.* **16**, 6903–6909 (2016).
11. May, V. & Kühn, O. *Charge and Energy Transfer Dynamics in Molecular Systems* (John Wiley & Sons, 2008).
12. Berkelbach, T. C., Hybertsen, M. S. & Reichman, D. R. Microscopic theory of singlet exciton fission. I. General formulation. *J. Chem. Phys.* **138**, 114102 (2013).
13. Tempelaar, R. & Reichman, D. R. Vibronic exciton theory of singlet fission. III. How vibronic coupling and thermodynamics promote rapid triplet generation in pentacene crystals. *J. Chem. Phys.* **148**, 244701 (2018).
14. Simpson, J. R. *et al.* Resonance Raman signature of intertube excitons in compositionally-defined carbon nanotube bundles. *Nat. Commun.* **9**, 637 (2018).
15. Lüer, L. *et al.* Coherent phonon dynamics in semiconducting carbon nanotubes: A quantitative study of electron-phonon coupling. *Phys. Rev. Lett.* **102**, 127401 (2009).
16. Mehlenbacher, R. D. *et al.* Energy transfer pathways in semiconducting carbon nanotubes revealed using two-dimensional white-light spectroscopy. *Nat. Commun.* **6**, 6732 (2015).
17. Michetti, P. & La Rocca, G. C. Exciton-phonon scattering and photoexcitation dynamics in J-aggregate microcavities. *Phys. Rev. B* **79**, 035325 (2009).
18. Maultzsch, J. *et al.* Phonon dispersion of carbon nanotubes. *Solid State Commun.* **121**, 471–474 (2002).
19. Xiang, B. *et al.* Two-dimensional infrared spectroscopy of vibrational polaritons. *Proc. Natl. Acad. Sci. U. S. A* **115**, 4845–4850 (2018).

20. Xiang, B. *et al.* State-selective polariton to dark state relaxation dynamics. *J. Phys. Chem. A* **123**, 5918–5927 (2019).

21. Schwartz, T. *et al.* Polariton dynamics under strong light–molecule coupling. *ChemPhysChem* **14**, 125–131 (2013).

22. Georgiou, K. *et al.* Generation of anti-Stokes fluorescence in a strongly coupled organic semiconductor microcavity. *ACS Photonics* **5**, 4343–4351 (2018).
